# ABR-DeepLabV3+: Adaptive Boundary-Refined Multi-Backbone DeepLabV3+ for MRI Brain Tumor Segmentation

**DOI:** 10.3390/bioengineering13101220

**Published:** 2026-10-19

**Authors:** Shoffan Saifullah, Rafał Dreżewski

**Affiliations:** 1 Faculty of Computer Science, AGH University of Krakow, 30-059 Krakow, Poland; 2 Department of Informatics, Universitas Pembangunan Nasional Veteran Yogyakarta, Yogyakarta 55281, Indonesia

**Keywords:** brain tumor segmentation, MRI, DeepLabV3+, Adaptive Boundary Refinement, Xception, multi-backbone evaluation, Dice Similarity Coefficient, Jaccard Index

## Abstract

Accurate brain tumor segmentation from magnetic resonance imaging (MRI) is essential for diagnosis, treatment planning, surgical guidance, and quantitative tumor assessment. However, automated segmentation remains challenging due to the heterogeneous tumor appearance, irregular morphology, weak tissue contrast, and ambiguous tumor boundaries. Conventional encoder–decoder segmentation models, including DeepLabV3+, can capture multi-scale semantic information but may still produce boundary inconsistencies when tumor margins are diffuse or visually similar to surrounding tissues. To address this limitation, this study proposes ABR-DeepLabV3+, an Adaptive Boundary-Refined Multi-Backbone DeepLabV3+ framework for MRI brain tumor segmentation. The proposed framework integrates an Adaptive Boundary Refinement (ABR) module after decoder fusion to enhance boundary-sensitive feature representation before final mask prediction. Four encoder backbones, namely InceptionResNetV2, Xception, MobileNetV2, and ResNet50, were evaluated under the same framework using the Figshare Brain Tumor Segmentation (FBTS) dataset consisting of 3064 MRI images from meningioma, glioma, and pituitary tumor classes. The evaluation employed patient-level train–validation–test partitioning together with patient-grouped five-fold cross-validation to reduce subject-level information leakage between data subsets. Experimental results show that ABR-DeepLabV3+ with Xception achieved the best overall test performance, with accuracy of 0.9994, Dice Similarity Coefficient (DSC) of 0.9753, and Jaccard Index (JI) of 0.9519. ResNet50 also achieved competitive performance with DSC of 0.9732 and JI of 0.9478, while MobileNetV2 demonstrated strong class-wise performance for meningioma and pituitary tumors. For the Xception configuration, the component-wise ablation analysis showed that Full ABR achieved higher performance than both plain DeepLabV3+ and an ordinary convolutional refinement control. Relative to plain DeepLabV3+, Full ABR increased DSC from 0.89002 to 0.97530 and JI from 0.80183 to 0.95190, while reducing the 95th-percentile Hausdorff Distance (HD95) from 8.72 to 3.28 pixels and the Average Symmetric Surface Distance (ASSD) from 2.41 to 0.87 pixels. Five-fold cross-validation showed that the Xception-based configuration achieved the highest mean DSC of 0.9017 and mean JI of 0.8258 among the evaluated backbones; however, the lower cross-validation mean relative to the single-split test result indicates sensitivity to patient-level data partitioning rather than unconditional evidence of robustness. Qualitative analysis using segmentation overlays, error heatmaps, and prediction-region heatmaps illustrated representative patterns of contour agreement and localized residual segmentation error without interpreting prediction-derived heatmaps as internal model attention or attribution. Overall, the findings indicate that the proposed ABR design improves within-dataset tumor-region overlap and boundary agreement relative to plain DeepLabV3+ under the evaluated experimental conditions, while external validation and repeated-run statistical evaluation remain necessary to establish broader generalizability and reproducibility.

## 1. Introduction

Brain tumors represent one of the most critical neurological disorders due to their aggressive progression, complex morphology, and substantial impact on patient survival and quality of life [1,2,3]. Accurate delineation of tumor regions from magnetic resonance imaging (MRI) is essential for diagnosis, treatment planning, radiotherapy guidance, surgical intervention, and longitudinal monitoring of disease progression [4,5,6]. MRI remains the preferred imaging modality for brain tumor analysis due to its superior soft-tissue contrast and the ability to visualize anatomical abnormalities across different tissue structures [7,8]. However, manual segmentation performed by radiologists is time-consuming, labor-intensive, and highly dependent on clinical expertise, particularly when tumor boundaries exhibit irregular shapes, heterogeneous intensities, edema regions, or low contrast against surrounding healthy tissues [9,10]. Consequently, the development of robust automated brain tumor segmentation frameworks has become an important research direction in medical image analysis.

Recent advances in deep learning have significantly improved medical image segmentation performance [4,11], particularly through convolutional neural network (CNN)-based architectures [12,13]. Among these approaches, U-Net and its variants have demonstrated strong effectiveness in biomedical segmentation tasks due to their encoder–decoder structure and skip-connection mechanisms that preserve spatial information [14,15,16]. Numerous extensions, including Attention U-Net, Residual U-Net, Dense U-Net, and Transformer-based segmentation frameworks, have further enhanced feature representation and contextual understanding [17,18,19,20,21,22,23,24,25,26,27]. Despite these advances, accurate tumor boundary delineation remains a major challenge because many segmentation networks primarily optimize overlap-based metrics such as Dice Similarity Coefficient (DSC) and Intersection over Union (IoU), while often neglecting fine-grained boundary preservation and edge consistency [28,29,30]. As a result, predicted tumor contours may exhibit blurred boundaries, discontinuities, or inaccurate localization near complex tumor edges.

DeepLabV3+ has emerged as a powerful semantic segmentation architecture owing to its Atrous Spatial Pyramid Pooling (ASPP) module and encoder–decoder design, which effectively capture multi-scale contextual information while preserving spatial details [31,32,33,34]. The ASPP mechanism enables the network to aggregate features from multiple receptive fields through dilated convolutions, allowing improved contextual understanding for objects of varying sizes [23,31]. Furthermore, the decoder component refines segmentation outputs by integrating low- and high-level feature representations [35]. Because of these capabilities, DeepLabV3+ has been successfully applied in numerous medical imaging tasks, including organ segmentation, lesion detection, and tumor analysis [36]. Nevertheless, conventional DeepLabV3+ frameworks still encounter limitations in preserving precise tumor boundaries, especially when tumors exhibit heterogeneous textures, irregular margins, or weak contrast transitions in MRI images [37].

Another important limitation observed in existing segmentation studies is the dependence on a single encoder backbone without investigating the robustness and generalization capability of different feature extraction architectures [8,14,38]. Different backbones possess distinct representational characteristics. For example, MobileNetV2 emphasizes computational efficiency through depthwise separable convolutions, ResNet50 benefits from residual learning for stable deep feature extraction, Xception provides enhanced spatial separability and multi-scale representation, while InceptionResNetV2 combines residual learning with multi-branch feature aggregation for richer contextual understanding [20,39,40,41,42,43,44]. However, the influence of these backbones on boundary-sensitive brain tumor segmentation remains inadequately explored, particularly within adaptive refinement-based segmentation frameworks.

Motivated by these limitations, this study argues that accurate MRI brain tumor segmentation requires not only strong multi-scale semantic representation, but also explicit refinement of tumor boundary regions [23,31,40,41,45]. In conventional DeepLabV3+ architectures, the decoder reconstructs the segmentation mask by combining high-level contextual features with low-level spatial details; however, this reconstruction process may still be insufficient when tumor margins are weak, irregular, or partially blended with surrounding tissue. Therefore, this study proposes ABR-DeepLabV3+, an Adaptive Boundary-Refined Multi-Backbone DeepLabV3+ framework designed to strengthen boundary-sensitive feature representation before the final segmentation prediction. The proposed Adaptive Boundary Refinement (ABR) module enhances local contour information through boundary-focused feature extraction and adaptive fusion, with the aim of reducing segmentation discrepancies near tumor edges while preserving the global tumor representation. In addition, multiple encoder backbones, including InceptionResNetV2, Xception, MobileNetV2, and ResNet50, are systematically evaluated to analyze how encoder selection is associated with segmentation accuracy, boundary agreement, and class-wise segmentation performance across meningioma, glioma, and pituitary tumors.

The proposed framework is evaluated using the Figshare Brain Tumor Segmentation (FBTS) dataset consisting of 3064 MRI images from three tumor categories, namely meningioma, glioma, and pituitary tumors. Extensive experiments are conducted using patient-level train–validation–test partitioning, patient-grouped five-fold cross-validation, quantitative overlap and boundary-distance evaluation, component-wise ablation, and qualitative visualization analysis. The evaluation includes Dice Similarity Coefficient (DSC), Jaccard Index (JI), Hausdorff Distance (HD), 95th-percentile Hausdorff Distance (HD95), Average Symmetric Surface Distance (ASSD), and accuracy measurements to assess both region-overlap performance and boundary agreement.

The main contributions of this study are summarized as follows:

1. We propose ABR-DeepLabV3+, an adaptive boundary-refined DeepLabV3+ framework designed to improve tumor contour preservation in MRI brain tumor segmentation.
2. We integrate an Adaptive Boundary Refinement (ABR) module to enhance boundary-sensitive feature representation before final mask prediction.
3. We conduct a multi-backbone evaluation using InceptionResNetV2, Xception, MobileNetV2, and ResNet50 to analyze the influence of encoder selection on segmentation performance.
4. We validate the proposed framework using patient-level train–validation–test partitioning, patient-grouped five-fold cross-validation, class-wise analysis, and qualitative visualization using error and prediction-region heatmaps.
5. We evaluate boundary agreement using Hausdorff Distance (HD), 95th-percentile Hausdorff Distance (HD95), and Average Symmetric Surface Distance (ASSD), in addition to DSC and JI, thereby distinguishing region-overlap performance from boundary-distance accuracy.
6. We conduct a component-wise ablation analysis of the local branch, dilated branches, adaptive gate, and final refinement convolution, together with an ordinary convolutional refinement control, to examine the contribution of the structured ABR design relative to conventional post-decoder convolutional refinement.

The remainder of this paper is organized as follows. Section 2 reviews related studies on brain tumor segmentation, DeepLabV3+, and boundary-aware segmentation methods. Section 3 describes in detail the proposed ABR-DeepLabV3+ framework. Section 4 presents the experimental setup, datasets, and evaluation metrics. Section 5 discusses the quantitative and qualitative results. Finally, Section 6 concludes the study and outlines future research directions.

## 2. Related Works

Automated brain tumor segmentation has become an important research direction in medical image analysis because accurate tumor delineation supports diagnosis, treatment planning, surgical guidance, and radiotherapy assessment. Early segmentation approaches commonly relied on handcrafted image processing techniques, including thresholding, region growing, clustering, edge detection, morphological operations, and texture-based feature extraction. Although these methods provided useful preliminary solutions, their performance was often sensitive to intensity variation, noise, tumor heterogeneity, and weak contrast between tumor and normal tissues. The irregular morphology of brain tumors, together with the diversity of MRI appearances across meningioma, glioma, and pituitary tumors, has motivated the development of more robust learning-based segmentation models.

### 2.1. CNN-Based Brain Tumor Segmentation

Convolutional neural networks (CNNs) have significantly advanced medical image segmentation by automatically learning hierarchical visual representations from imaging data [4,46]. Compared to handcrafted methods, CNN-based models can extract complex spatial, textural, and semantic features directly from MRI images, making them more suitable for handling heterogeneous tumor regions [47,48]. U-Net has become one of the most widely used architectures in biomedical segmentation due to its encoder–decoder structure and skip connections, which combine high-level semantic information with low-level spatial details [8,49,50]. Several U-Net variants, including Residual U-Net, Attention U-Net, Dense U-Net, and hybrid encoder-decoder models, have been introduced to improve feature propagation, localization accuracy, and robustness against anatomical variability [20,21,22,23,24,25,38].

Despite their effectiveness, many CNN-based segmentation models still face challenges when dealing with ambiguous tumor boundaries [51,52]. Overlap-based optimization can produce high Dice or Jaccard scores while still generating imperfect contours, especially near irregular tumor margins [27]. This issue is clinically relevant because boundary errors can affect volumetric estimation, surgical planning, and treatment response monitoring [53]. Therefore, recent studies have increasingly emphasized not only region-level segmentation accuracy but also boundary-aware refinement and contour consistency.

Beyond conventional convolutional architectures, recent neural-network research has also explored alternative representation mechanisms such as Kolmogorov–Arnold Networks (KANs), which replace fixed activation functions with learnable function parameterizations and have demonstrated effective nonlinear predictive modeling in other engineering domains [54]. Although such approaches are not direct medical-image segmentation comparators, they illustrate the broader trend toward more adaptive neural representation mechanisms.

### 2.2. DeepLabV3+ and Transfer Learning in Medical Image Segmentation

DeepLabV3+ is a semantic segmentation architecture that has attracted attention in medical image analysis due to its ability to capture multi-scale contextual information through Atrous Spatial Pyramid Pooling (ASPP) [32,33,37,40,41]. By applying atrous convolutions with different dilation rates, ASPP enables the network to analyze objects at multiple receptive fields without excessively increasing the number of parameters. This capability is particularly useful in brain tumor segmentation, because tumor regions may vary significantly in size, shape, texture, and spatial distribution. The decoder component of DeepLabV3+ further improves segmentation localization by combining deep semantic features with low-level spatial features.

Transfer learning has also played an important role in improving segmentation performance, especially when annotated medical datasets are limited [55]. Pre-trained encoder backbones such as ResNet, Xception, MobileNet, and Inception-based networks provide rich feature representations learned from large-scale image datasets [56]. These features can be adapted to medical image segmentation tasks through fine-tuning [57]. ResNet-based encoders benefit from residual connections that improve gradient flow and training stability [35,58], while Xception and MobileNetV2 employ depthwise separable convolutions that reduce computational cost and improve feature efficiency [59]. InceptionResNetV2 combines residual learning with multi-branch feature extraction, enabling richer contextual representation.

Previous studies have shown that DeepLabV3+ can be effective for MRI-based brain tumor segmentation [31,60]. DeepLabV3+ with ResNet18 demonstrated strong pixel-wise segmentation performance on the Figshare Brain Tumor Segmentation dataset, confirming the feasibility of CNN-transfer learning for tumor localization [45]. Subsequent work using DeepLabV3+ with an Xception encoder further improved segmentation performance by exploiting depthwise separable convolution and multi-scale feature extraction [41]. However, these studies focused primarily on individual backbone configurations. A systematic investigation of how different encoder backbones behave under an adaptive boundary-refined DeepLabV3+ framework remains limited.

### 2.3. Boundary-Aware Segmentation and Refinement Strategies

Boundary preservation is a critical requirement in medical image segmentation because small contour deviations may lead to inaccurate tumor volume estimation and reduced clinical reliability [1,61,62]. Conventional segmentation models often prioritize region overlap but may not explicitly enforce boundary consistency [63]. To address this limitation, boundary-aware strategies have been introduced in several forms, including edge-guided refinement, contour-aware loss functions, attention-based boundary modules, and multi-scale decoder enhancement [64,65]. These approaches aim to improve segmentation performance by strengthening the representation of transition regions between tumor and non-tumor tissues.

Boundary-aware refinement is particularly relevant for brain tumor MRI because tumor margins may be diffuse, irregular, or visually similar to adjacent anatomical structures [61,66]. Glioma regions, for example, may exhibit infiltrative and heterogeneous patterns, while meningioma and pituitary tumors may present different boundary characteristics depending on location and contrast distribution [1]. A segmentation framework that can refine edge-sensitive features before final mask prediction may therefore improve both quantitative overlap metrics and qualitative contour accuracy [67]. However, many existing methods treat boundary refinement as an auxiliary post-processing step or integrate it without systematically analyzing its interaction with different encoder backbones [68,69].

Recent studies further demonstrate that segmentation performance can be improved by modifying either the input localization strategy or the manner in which boundary information is incorporated into the segmentation network. Nefediev et al. [70] investigated cascaded detection and segmentation for prostate cancer on T2-weighted MRI, combining prostate localization with subsequent region-focused segmentation. Their results showed that restricting segmentation to a localized anatomical region can reduce irrelevant background information and improve lesion delineation. Although their approach addresses prostate rather than brain tumor MRI and relies on a multi-stage detection–segmentation pipeline, it illustrates the continuing importance of spatially focused feature processing in medical image segmentation.

More directly related to boundary-aware brain tumor segmentation, Gu et al. [71] proposed Bridge-Net, which combines a structural boundary prior with prediction-derived ambiguity and introduces the resulting cue into encoder–decoder skip features through gated residual injection. This strategy restricts feature correction primarily to regions that are simultaneously boundary-like and prediction-ambiguous. In contrast, the proposed ABR-DeepLabV3+ performs refinement after DeepLabV3+ decoder fusion through parallel local and dilated boundary branches followed by an adaptive gate. Thus, Bridge-Net modifies multi-level skip-feature transfer using boundary–ambiguity guidance, whereas the present framework refines the already-decoded representation using local and multi-scale contextual information. In addition, the present study evaluates the same refinement design across four encoder backbones and includes an ordinary-convolution control and component-wise ablation to separate the contribution of the structured refinement mechanism from that of additional post-decoder convolutional processing.

### 2.4. Research Gap and Position of This Study

Although CNN-based and DeepLabV3+-based models have achieved strong results in brain tumor segmentation [4,23,60], several gaps remain. First, many studies evaluate only a single encoder backbone, making it difficult to determine whether performance gains are caused by the segmentation framework, the selected encoder, or dataset-specific characteristics [8,14,38]. Second, standard DeepLabV3+ architectures rely mainly on ASPP and decoder fusion, which may not be sufficient to preserve fine tumor boundaries in ambiguous MRI regions [32,33,37]. Third, segmentation performance is frequently emphasized through region-overlap measures, whereas explicit boundary-distance evaluation and qualitative analysis of residual contour errors provide complementary information about segmentation quality [28,29,30]. Moreover, the combined evaluation of multiple encoder backbones, boundary-sensitive metrics, and controlled component-wise ablation within a common DeepLabV3+-based framework remains comparatively limited.

To address these gaps, this study proposes ABR-DeepLabV3+, an Adaptive Boundary-Refined Multi-Backbone DeepLabV3+ framework for MRI brain tumor segmentation. The proposed method integrates an Adaptive Boundary Refinement module into DeepLabV3+ to enhance contour-sensitive feature representation before final mask prediction. In addition, four encoder backbones, namely InceptionResNetV2, Xception, MobileNetV2, and ResNet50, are evaluated under the same segmentation framework to analyze the influence of backbone selection on accuracy, Dice Similarity Coefficient, Jaccard Index, boundary-distance measures, and class-wise segmentation performance. The evaluation further incorporates patient-level data partitioning, five-fold cross-validation, HD95 and ASSD boundary analysis, qualitative error and prediction-region visualization, and a component-wise ablation study including an ordinary convolutional refinement control. By combining boundary refinement, multi-backbone evaluation, cross-validation, boundary-distance assessment, controlled ablation, and qualitative visualization, this study provides a more comprehensive evaluation of DeepLabV3+-based brain tumor segmentation on the FBTS dataset.

## 3. Proposed Method

This section describes the proposed ABR-DeepLabV3+ framework for MRI brain tumor segmentation. The proposed method extends the conventional DeepLabV3+ architecture by integrating an Adaptive Boundary Refinement (ABR) module before the final segmentation prediction. The main objective of this module is to strengthen the boundary-sensitive feature representation and reduce segmentation errors around irregular tumor margins. In addition, the framework is evaluated with multiple encoder backbones to examine how different feature extraction mechanisms are associated with tumor localization, boundary agreement, and class-wise segmentation performance.

### 3.1. Overview of the ABR-DeepLabV3+ Framework

**Figure 1:**
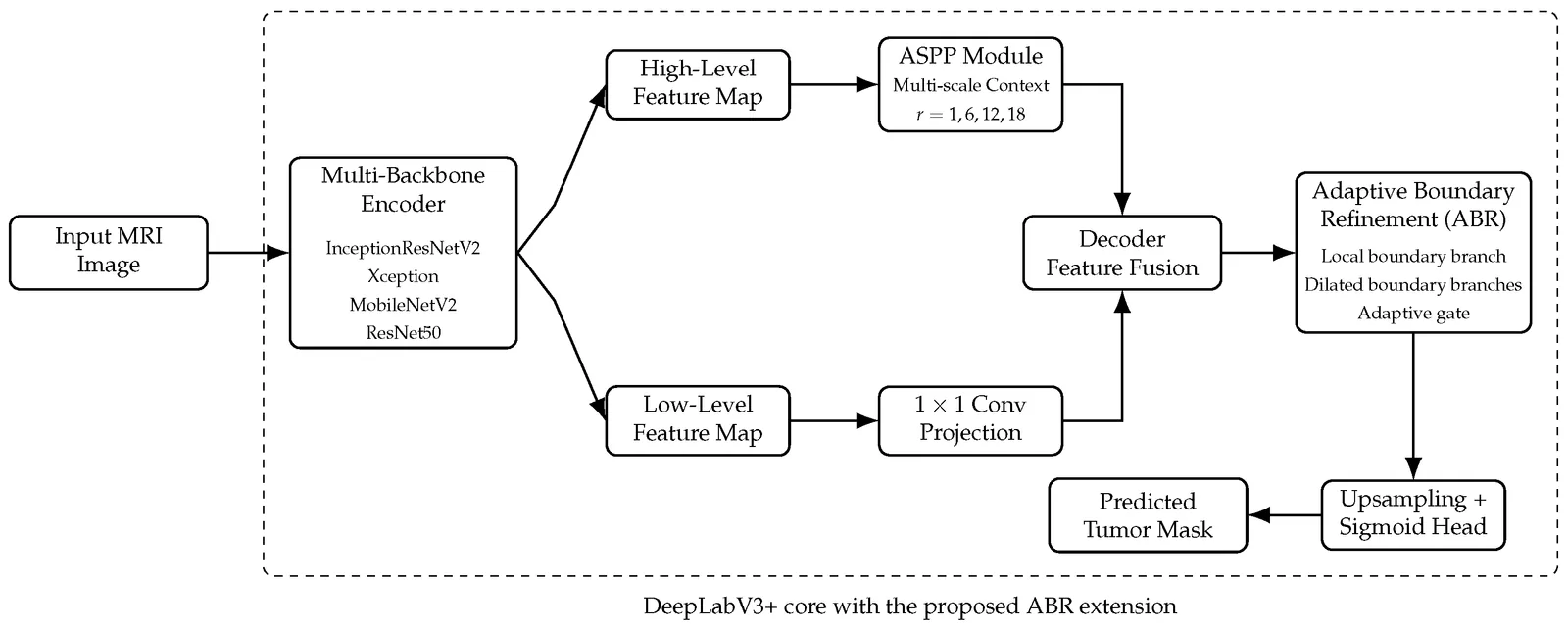
Overall architecture of the proposed ABR-DeepLabV3+ framework. The input MRI image is processed by a selected encoder backbone to extract low-level and high-level features. The high-level features are passed through the ASPP module to capture multi-scale contextual information, while low-level features are projected using a 1×1 convolution for spatial detail preservation. The decoder fuses both feature levels, followed by the proposed Adaptive Boundary Refinement (ABR) module to enhance tumor boundary representation before final binary mask prediction.

The proposed ABR-DeepLabV3+ framework follows an encoder–ASPP–decoder–refinement structure [40,41], as illustrated in Figure 1. Given an input MRI image $I\in R^{H\times W\times 3}$, the encoder first extracts hierarchical feature representations from low-level spatial patterns to high-level semantic information. In this study, four encoder backbones are investigated, namely InceptionResNetV2, Xception, MobileNetV2, and ResNet50. The use of multiple backbones enables a controlled evaluation of how different feature extraction mechanisms affect segmentation accuracy and boundary preservation.

The high-level feature map generated by the encoder is processed by the Atrous Spatial Pyramid Pooling (ASPP) module to capture multi-scale contextual information using parallel atrous convolutions with different dilation rates. Meanwhile, low-level features from earlier encoder layers are projected using a 1×1 convolution to preserve spatial details while reducing channel dimensionality. The ASPP-enhanced high-level features and projected low-level features are then fused in the decoder stage to recover spatial resolution and improve tumor localization.

Unlike the standard DeepLabV3+ decoder, the proposed framework introduces an Adaptive Boundary Refinement (ABR) module after decoder fusion. This module is designed to refine the reconstructed feature map by emphasizing local boundary cues and dilated contextual boundary information. The refined feature representation is subsequently upsampled to the original image resolution and passed through a final convolutional layer with sigmoid activation to produce the predicted binary tumor mask. By inserting the ABR module before the final prediction layer, the proposed framework explicitly strengthens the representation of boundary-sensitive features while preserving the global tumor structure.

The overall mapping of the proposed framework can be expressed as:

(1) $$\hat{Y}=S\left(R_{ABR}\left(D\left(A\left(E_{b}\left(I\right)\right),L_{b}\left(I\right)\right)\right)\right),$$

where $\hat{Y}$ denotes the predicted tumor mask, $E_{b}\left(\cdot \right)$ represents the encoder backbone, A(·) denotes the ASPP module, $L_{b}\left(\cdot \right)$ represents the low-level feature projection from the encoder, D(·) denotes the decoder fusion process, $R_{ABR}\left(\cdot \right)$ represents the proposed Adaptive Boundary Refinement module, and S(·) denotes the final segmentation layer.

### 3.2. Multi-Backbone Encoder Design

**Figure 2:**
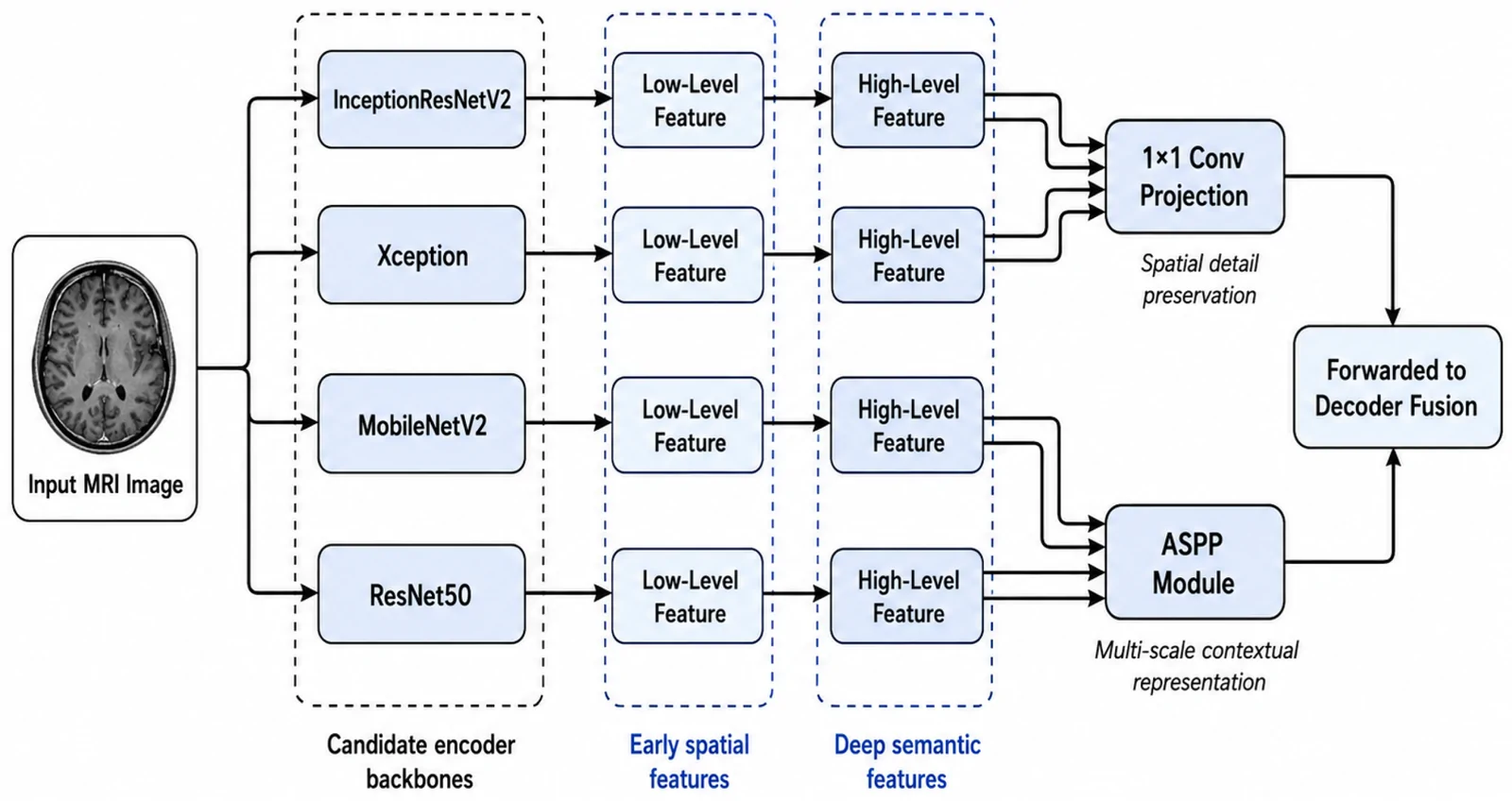
Conceptual illustration of the multi-backbone encoder design in ABR-DeepLabV3+. Four encoder backbones are evaluated, namely InceptionResNetV2, Xception, MobileNetV2, and ResNet50. For each backbone, an early low-level feature map is selected for spatial detail preservation and projected through a 1×1 convolution, while a deeper high-level feature map is selected for ASPP-based multi-scale contextual processing.

The encoder is responsible for extracting hierarchical image features that determine the quality of subsequent segmentation reconstruction [14,72]. In this study, four encoder backbones are investigated—InceptionResNetV2, Xception, MobileNetV2, and ResNet50 [73,74,75]—as illustrated in Figure 2. These architectures were selected because they represent different design principles in deep feature extraction and provide a diverse basis for evaluating the effectiveness of the proposed Adaptive Boundary Refinement strategy.

ResNet50 employs residual connections to improve gradient propagation and enable stable deep feature learning. This makes it suitable as a strong residual baseline for segmentation tasks. MobileNetV2 uses inverted residual blocks and depthwise separable convolutions, providing a lightweight feature extractor with reduced computational complexity. Xception extends the idea of depthwise separable convolution and provides efficient spatial-channel decomposition, which can improve feature discrimination while maintaining computational efficiency. InceptionResNetV2 combines multi-branch Inception-style feature extraction with residual learning, allowing the network to capture richer contextual information across different receptive fields [20,39,40,41,42,43,44].

For each backbone, a high-level feature map is selected for ASPP processing, while an earlier low-level feature map is used for spatial detail preservation [23,76]. The high-level feature map contains rich semantic information but has reduced spatial resolution, whereas the low-level feature map retains finer spatial details useful for boundary reconstruction. As depicted in Figure 2, the low-level feature is forwarded to a 1×1 projection layer, while the high-level feature is forwarded to the ASPP module. This dual-feature strategy allows the network to combine semantic context with fine spatial structure during decoder fusion.

The low-level feature map $F_{low}$ is projected using a 1×1 convolution:

(2) $$F_{low}^{\prime }=\phi_{1\times 1}\left(F_{low}\right),$$

where $\phi_{1\times 1}\left(\cdot \right)$ denotes a 1×1 convolution followed by batch normalization and ReLU activation. This operation reduces the number of channels and prevents low-level features from dominating the fused representation. In contrast, the high-level feature map $F_{high}$ is processed by the ASPP module to enhance multi-scale contextual representation before decoder fusion. By jointly exploiting $F_{low}^{\prime }$ and the ASPP-enhanced $F_{high}$, the proposed framework can preserve boundary detail while maintaining strong semantic discrimination across different tumor appearances.

### 3.3. Atrous Spatial Pyramid Pooling

**Figure 3:**
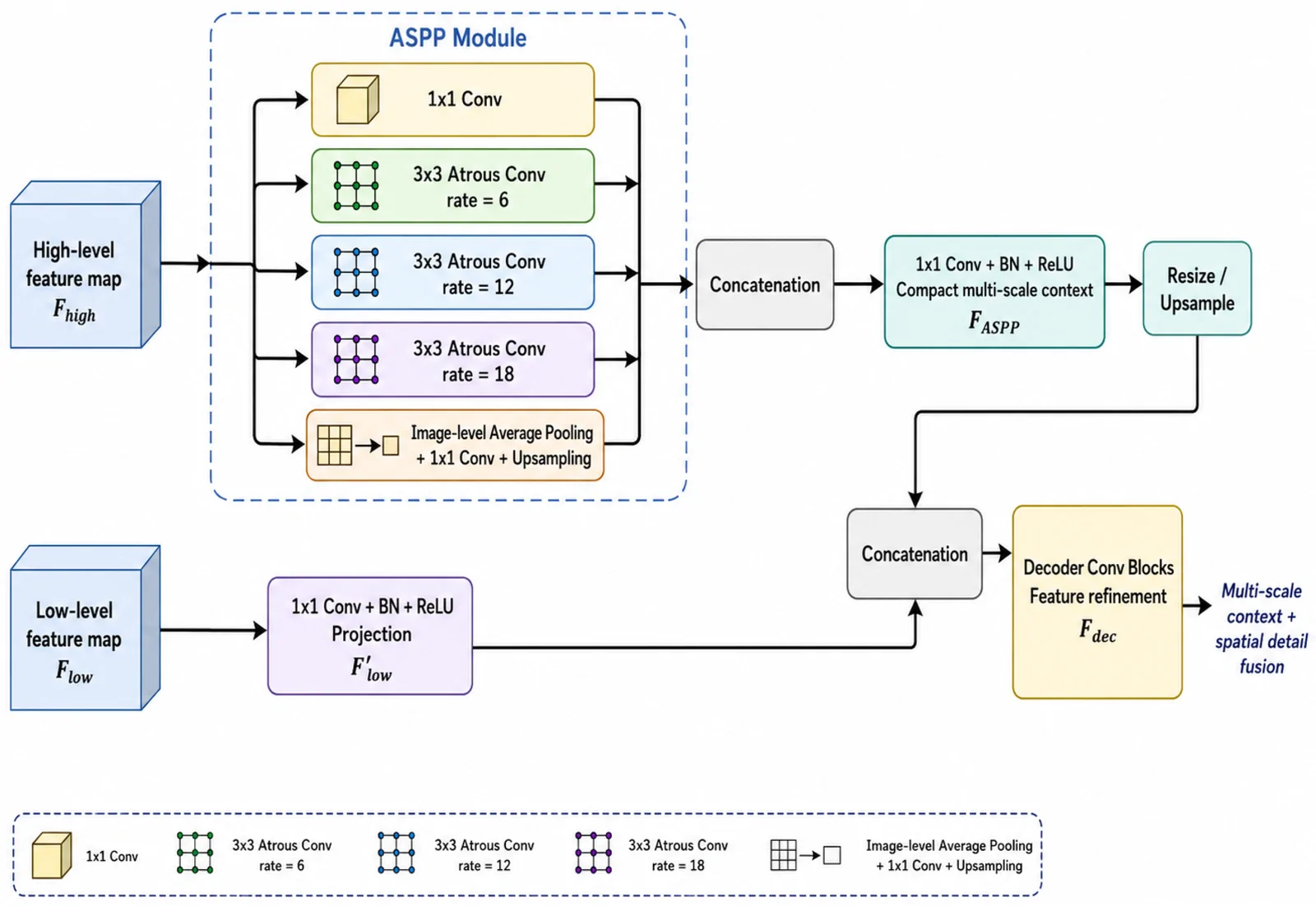
Visualization of the ASPP and decoder fusion process in the proposed ABR-DeepLabV3+ framework. The high-level feature map $F_{high}$ is processed by parallel ASPP branches, including 1×1 convolution, atrous convolutions with dilation rates of 6, 12, and 18, and image-level average pooling. The concatenated ASPP output is compressed into $F_{ASPP}$ and resized before being fused with the projected low-level feature map $F_{low}^{\prime }$ for decoder refinement.

The Atrous Spatial Pyramid Pooling (ASPP) module is used to extract multi-scale contextual features from the high-level encoder output [77,78], as illustrated in Figure 3. Brain tumors may vary substantially in size, shape, texture, and spatial distribution; therefore, a single receptive field is often insufficient for robust segmentation. In MRI brain tumor segmentation, small tumors require preservation of local spatial details, whereas larger and heterogeneous tumor regions require broader contextual understanding. ASPP addresses this limitation by applying multiple parallel convolutional branches with different dilation rates, allowing the network to capture both local and global contextual information from the same high-level feature representation [32,33,37,40,41].

Given the high-level feature map $F_{high}$ extracted from the encoder backbone, the ASPP module processes this feature map through five parallel branches: a 1×1 convolution branch, three 3×3 atrous convolution branches with dilation rates of 6, 12, and 18, and an image-level average pooling branch. The 1×1 convolution captures local channel-wise feature interactions, while the atrous convolution branches expand the effective receptive field without reducing the spatial resolution of the feature map. The image-level pooling branch further introduces global contextual information by summarizing the entire feature map before upsampling it back to the original ASPP feature resolution.

The ASPP output is computed as:

(3) $$\begin{array}{cc} F_{ASPP}=\phi_{1\times 1}([ & \phi_{1\times 1}\left(F_{high}\right),\phi_{3\times 3}^{r=6}\left(F_{high}\right),\\ & \phi_{3\times 3}^{r=12}\left(F_{high}\right),\\ & \phi_{3\times 3}^{r=18}\left(F_{high}\right),\phi_{pool}\left(F_{high}\right)]), \end{array}$$

where $\phi_{3\times 3}^{r}\left(\cdot \right)$ denotes a 3×3 atrous convolution with dilation rate *r*, $\phi_{pool}\left(\cdot \right)$ represents image-level average pooling followed by 1×1 convolution and upsampling, and [·] denotes channel-wise concatenation. The final 1×1 convolution compresses the concatenated multi-scale features into a compact contextual representation. This operation reduces feature redundancy while preserving contextual information from different receptive fields.

After ASPP processing, $F_{ASPP}$ is resized to match the spatial resolution of the projected low-level feature map $F_{low}^{\prime }$. The resized ASPP feature contains rich semantic and multi-scale contextual information, whereas $F_{low}^{\prime }$ preserves fine spatial details from earlier encoder layers. These two feature maps are concatenated and processed by decoder convolutional blocks to produce the decoded representation:

(4) $$F_{dec}=\phi_{dec}\left([Resize\left(F_{ASPP}\right),F_{low}^{\prime }]\right),$$

where $F_{dec}$ represents the decoded feature map and $\phi_{dec}\left(\cdot \right)$ denotes successive convolutional refinement operations. This fusion enables the decoder to combine high-level tumor semantics with low-level spatial detail, which is essential for accurate tumor localization and subsequent boundary refinement by the ABR module.

### 3.4. Adaptive Boundary Refinement Module

**Figure 4:**
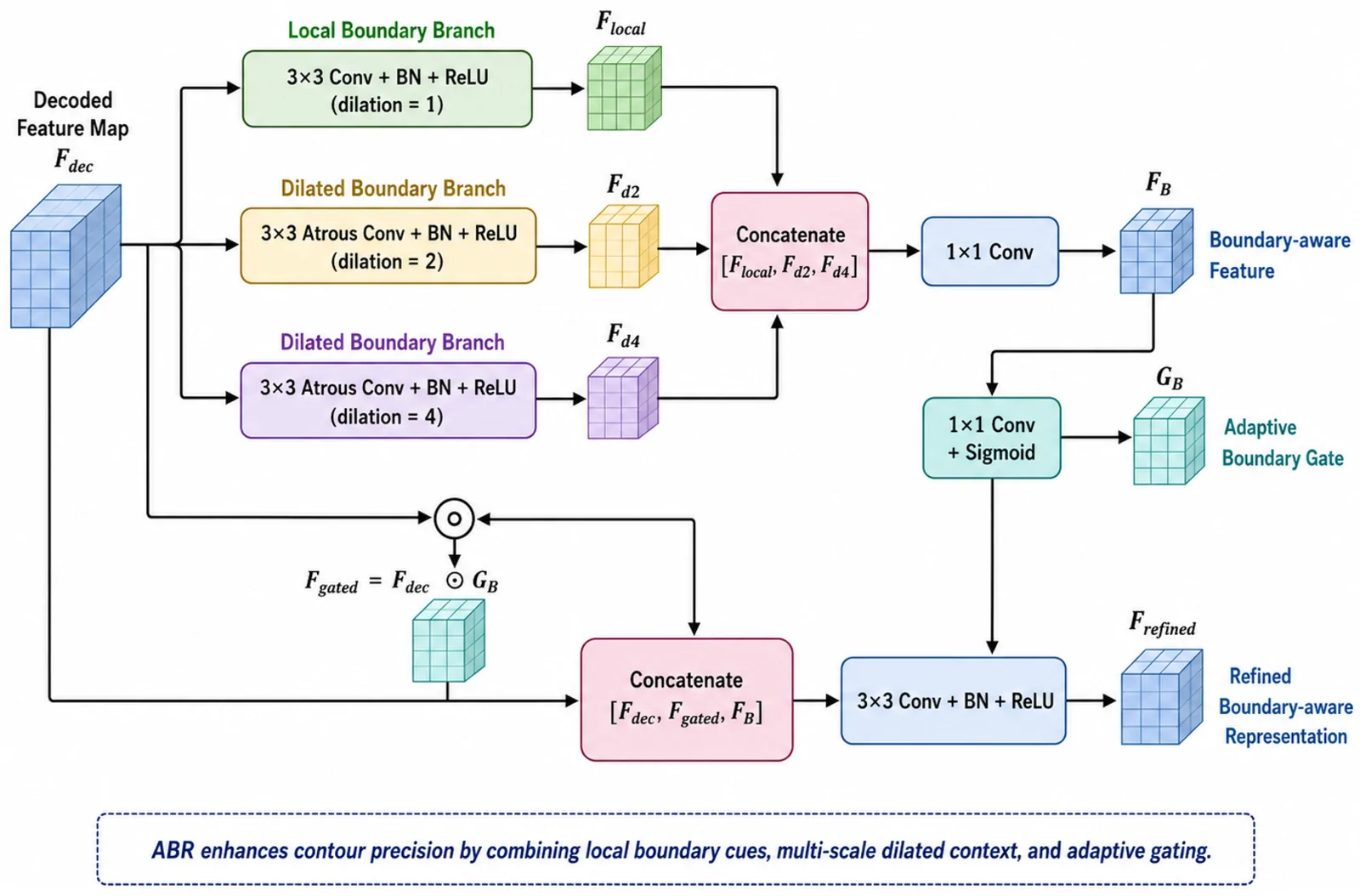
Architecture of the proposed Adaptive Boundary Refinement (ABR) module. The decoded feature map $F_{dec}$ is processed through a local boundary branch and two dilated boundary branches with dilation rates 2 and 4. The extracted boundary-aware features are fused to produce $F_{B}$, followed by a sigmoid-based adaptive gate $G_{B}$ that modulates the decoded representation. The original decoded feature, gated feature, and fused boundary feature are then concatenated and refined to generate the final boundary-enhanced representation $F_{refined}$.

Although the decoder in DeepLabV3+ improves spatial reconstruction, segmentation errors may still occur around tumor boundaries due to weak contrast [28,29,30,37,51], irregular morphology, blurred contour transitions, and heterogeneous texture patterns. These issues are particularly relevant in brain tumor MRI, where the boundary between tumor tissue and surrounding anatomical structures may not be sharply defined. To address this limitation, the proposed Adaptive Boundary Refinement (ABR) module is introduced after decoder feature fusion, as illustrated in Figure 4. The purpose of this module is to explicitly enhance the boundary-sensitive feature learning while preserving the global tumor structure represented in the decoded feature map.

The ABR module enhances boundary information through three complementary components: a local boundary branch, a dilated boundary context branch, and an adaptive fusion gate. The local branch focuses on fine contour details, whereas the dilated branches capture broader boundary continuity and contextual structure at multiple receptive fields. The adaptive gate then learns how strongly boundary-aware information should contribute to the refined representation.

Given the decoded feature map $F_{dec}$, the local boundary branch extracts fine contour information using a standard convolution:

(5) $$F_{local}=\phi_{3\times 3}^{r=1}\left(F_{dec}\right),$$

where $\phi_{3\times 3}^{r=1}\left(\cdot \right)$ denotes a standard 3×3 convolution followed by normalization and nonlinear activation. This branch is intended to preserve short-range spatial detail and local edge structure.

To capture wider boundary continuity and contextual interactions, two additional dilated convolution branches are introduced with dilation rates of 2 and 4:

(6) $$F_{d2}=\phi_{3\times 3}^{r=2}\left(F_{dec}\right),$$

(7) $$F_{d4}=\phi_{3\times 3}^{r=4}\left(F_{dec}\right),$$

where $\phi_{3\times 3}^{r=2}\left(\cdot \right)$ and $\phi_{3\times 3}^{r=4}\left(\cdot \right)$ represent atrous convolutions with dilation rates 2 and 4, respectively. These branches allow the model to aggregate boundary-related context over a larger spatial range without reducing the spatial resolution of the feature map.

The local and dilated boundary features are then concatenated and fused through a 1×1 convolution:

(8) $$F_{B}=\phi_{1\times 1}\left([F_{local},F_{d2},F_{d4}]\right),$$

where $F_{B}$ denotes the fused boundary-aware feature representation, and [·] indicates channel-wise concatenation. This fusion operation integrates complementary information from local and multi-scale boundary responses into a compact representation.

To adaptively control the influence of the extracted boundary features, a sigmoid gating mechanism is applied:

(9) $$G_{B}=\sigma \left(\phi_{1\times 1}\left(F_{B}\right)\right),$$

where $G_{B}$ is the adaptive boundary gate and σ(·) denotes the sigmoid activation function. The gate learns a spatial weighting map that highlights boundary-relevant responses while suppressing less informative regions.

The decoded feature map is then modulated through element-wise multiplication with the learned gate:

(10) $$F_{gated}=F_{dec}⊙G_{B},$$

where ⊙ denotes element-wise multiplication. This gating process allows the network to selectively strengthen feature responses around tumor contours while preserving the original semantic structure.

Finally, the original decoded feature, the gated feature, and the fused boundary-aware feature are concatenated and refined using a final convolutional operation:

(11) $$F_{refined}=\phi_{3\times 3}\left([F_{dec},F_{gated},F_{B}]\right).$$

The resulting feature map $F_{refined}$ serves as the boundary-enhanced representation that is subsequently passed to the final segmentation layer. This design allows the model to preserve global tumor structure while explicitly improving local contour consistency and boundary precision. Because the ABR module is placed after decoder fusion, it can be integrated with different encoder backbones without requiring modification of the encoder architecture. This makes it well suited for the multi-backbone evaluation performed in this study.

### 3.5. Segmentation Head

After Adaptive Boundary Refinement, the refined feature map $F_{refined}$ contains both high-level tumor semantics and boundary-enhanced spatial information. This feature map is upsampled to the original image resolution using bilinear interpolation to ensure pixel-wise alignment with the input MRI image. A final 1×1 convolution followed by sigmoid activation is then applied to convert the refined feature representation into a binary tumor probability map:

(12) $$\hat{Y}=\sigma \left(\phi_{1\times 1}\left(Resize\left(F_{refined}\right)\right)\right),$$

where Resize(·) denotes bilinear upsampling, $\phi_{1\times 1}\left(\cdot \right)$ represents the final point-wise convolution, and σ(·) denotes the sigmoid activation function. The output $\hat{Y}\in {\left[0,1\right]}^{H\times W}$ represents the pixel-wise probability of belonging to the tumor region. A value close to 1 indicates high confidence for the tumor class, whereas a value close to 0 indicates background tissue.

During inference, the probability map is converted into a binary segmentation mask using a threshold of 0.5:

(13) $$Y_{pred}\left(i,j\right)=\left\{\begin{array}{cc} 1, & \hat{Y}\left(i,j\right)\geq 0.5,\\ 0, & \hat{Y}\left(i,j\right)<0.5. \end{array}\right.$$

where $Y_{pred}\left(i,j\right)$ denotes the predicted class label at pixel location (i,j). This thresholding step produces the final tumor mask used for quantitative evaluation and qualitative visualization. Since the task is formulated as binary tumor segmentation, the sigmoid-based segmentation head is appropriate for distinguishing tumor pixels from background pixels.

### 3.6. Loss Function

The proposed framework is trained using binary cross-entropy (BCE) as the optimization loss [23,31]. Binary cross-entropy encourages pixel-wise classification accuracy by penalizing discrepancies between the predicted tumor probabilities and the corresponding binary ground-truth labels. The binary cross-entropy loss is defined as:

(14) $$L_{BCE}=-\frac{1}{N}\sum_{i=1}^{N}\left[Y_{i}\log\left({\hat{Y}}_{i}\right)+\left(1-Y_{i}\right)\log\left(1-{\hat{Y}}_{i}\right)\right],$$

where $Y_{i}$ and ${\hat{Y}}_{i}$ represent the ground-truth binary label and predicted tumor probability for pixel *i*, respectively, and *N* is the total number of pixels.

The BCE loss was used directly as the training objective for all evaluated backbone, baseline, control, and ablation configurations. The Dice Similarity Coefficient (DSC) and Jaccard Index (JI) were used as segmentation-performance measures rather than as components of the optimization objective. Their definitions and evaluation procedures are provided in Section 4.6. This distinction ensures consistency between the implemented optimization objective and the loss values reported in the quantitative results.

## 4. Experimental Setup

This section describes the dataset, the preprocessing strategy, the training configuration, the validation protocol, and the evaluation metrics used to assess the proposed ABR-DeepLabV3+ framework. The experiments were designed to evaluate both the segmentation performance and the performance consistency of different encoder backbones under the same adaptive boundary-refined DeepLabV3+ framework.

### 4.1. Dataset Description

The experiments were conducted using the Figshare Brain Tumor Segmentation (FBTS) dataset [79], which contains 3064 brain MRI images with the corresponding ground-truth tumor masks. The dataset includes three tumor categories: meningioma, glioma, and pituitary tumors. Specifically, the dataset consists of 708 meningioma images, 1426 glioma images, and 930 pituitary tumor images. These three tumor types represent different anatomical appearances and segmentation challenges. Meningioma cases often present relatively compact tumor structures with distinguishable boundaries, and glioma cases may show more heterogeneous and irregular regions, while pituitary tumors can vary in size and location depending on their anatomical position.

In this study, the segmentation task was formulated as a binary tumor segmentation problem, where each pixel was classified as tumor or background. Although the dataset contains three tumor categories, the model was trained to delineate tumor regions from non-tumor tissues. The class-wise evaluation was then performed separately for meningioma, glioma, and pituitary cases to examine variation in segmentation performance across the three tumor categories.

### 4.2. Image Preprocessing

All MRI images and the corresponding masks were resized to 256×256 pixels to ensure a consistent input resolution in all experiments. Since the original images were processed as grayscale MRI slices, each image was replicated into three channels to match the input requirement of ImageNet-pretrained encoder backbones. The image intensity values were normalized to the range [0, 1] by dividing the pixel values by 255. The ground-truth masks were resized using nearest-neighbor interpolation to preserve the consistency of the binary label. The masks were then binarized, where tumor pixels were assigned a value of 1 and background pixels a value of 0.

The preprocessing pipeline can be summarized as follows: image resizing, grayscale-to-three-channel conversion, intensity normalization, mask resizing, and binary mask conversion. This procedure ensures that all backbones receive identical input data and that performance differences arise from architectural behavior rather than preprocessing variation.

### 4.3. Training, Validation, and Testing Protocol

The dataset was divided into training, validation, and testing subsets using an approximately 80:10:10 split. To prevent information leakage between MRI slices originating from the same subject, the partitioning was performed at the patient level rather than at the individual image level. All slices belonging to a given patient were assigned exclusively to one subset, ensuring that no patient contributed images to more than one of the training, validation, or testing partitions. The training subset was used to optimize model parameters, the validation subset was used to monitor model performance during training, and the testing subset was held out for final performance evaluation. The held-out test subset was not used during training, validation, or model selection and was evaluated only after the final model had been selected. The same split configuration was used for all backbone variants to ensure fair comparison. The same patient-level partitions were also used for the corresponding plain DeepLabV3+ and ABR-DeepLabV3+ configurations.

In addition to the train–validation–test evaluation, five-fold cross-validation was performed to assess performance consistency and sensitivity to patient-level data partitioning. The cross-validation procedure was performed on the development cohort using patient identifiers as grouping variables, while the independent held-out test cohort remained excluded from all cross-validation folds. In each fold, the data was partitioned into training and validation subsets and the model was evaluated using the same performance metrics. All MRI slices belonging to the same patient were retained within the same fold, thereby preventing patient overlap between the corresponding training and validation partitions. Each fold was treated as an independent experiment: the model was reconstructed and its trainable parameters were newly initialized before training, and neither model weights nor optimizer states were transferred between folds. The cross-validation results were used to examine the consistency of model performance across different patient-level partitions and to assess the extent to which the observed performance depended on the particular data split. Patient identifiers were programmatically checked before training to verify the absence of overlap between the training, validation, and testing subsets and between the training and validation partitions of each cross-validation fold.

### 4.4. Implementation Details

The proposed ABR-DeepLabV3+ framework was implemented using TensorFlow and Keras. All models were trained using the Adam optimizer with an initial learning rate of $1\times 10^{-4}$. The maximum number of training epochs was set to 50. The batch size was fixed at 8 for the main train–validation–test experiments. To improve reproducibility, a random seed of 42 was consistently applied to Python 3.9.5, NumPy 2.0.2, TensorFlow 2.19.0, and the patient-level data-partitioning procedure. The learning rate was kept fixed during training, and the models were trained for the predefined maximum number of epochs without an adaptive learning-rate scheduler or early-stopping criterion.

The encoder backbones were initialized using ImageNet-pretrained weights and remained trainable during optimization. No online data augmentation was applied; consequently, all compared models received the same preprocessed MRI images without stochastic geometric or intensity transformations.

The experiments were conducted using NVIDIA A100-SXM4 GPUs (NVIDIA Corporation, Santa Clara, CA, USA) with 40 GB memory, accessed through the PLGrid infrastructure at ACK Cyfronet AGH (Kraków, Poland). The same training configuration, loss function, preprocessing pipeline, and evaluation protocol were applied to all encoder backbones. For the main train–validation–test experiments, the same patient-level data partitions, random-seed configuration, batch size, learning rate, and training duration were used for all backbone variants. This controlled setting allowed the effect of backbone selection and Adaptive Boundary Refinement to be analyzed consistently. To ensure a fair comparison, the same training configuration was applied to both the plain DeepLabV3+ baselines and the proposed ABR-DeepLabV3+ variants; for the direct plain-versus-Full-ABR comparison, the principal architectural difference was the inclusion of the ABR module after decoder fusion. The ordinary convolutional refinement control and the component-removal variants described in the ablation study were trained under the same experimental protocol while modifying only the refinement configuration under investigation.

For the ablation experiments, the ordinary convolutional refinement control replaced the complete ABR module with a single standard 3×3 convolutional refinement layer operating on the decoder feature map $F_{\mathrm{dec}}$. The layer used 256 input channels and 256 output channels, a stride of 1, dilation rate of 1, and same spatial padding, followed by batch normalization and ReLU activation. Thus, the control preserved the spatial resolution and channel dimensionality of the decoder representation while removing the local/dilated multi-branch processing and adaptive gating used by the complete ABR module.

For the “ABR w/o Adaptive Gate” configuration, the sigmoid gate $G_{B}$ and the element-wise modulation $F_{\mathrm{gated}}=F_{\mathrm{dec}}⊙G_{B}$ were removed. The fused boundary representation $F_{B}$ was instead concatenated directly with the original decoder feature, and the resulting representation was processed by the final refinement convolution. Accordingly, this variant can be expressed as

(15) $$F_{\mathrm{refined}}^{\left(-\mathrm{gate}\right)}=\phi_{3\times 3}\left([F_{\mathrm{dec}},F_{B}]\right).$$

For the “ABR w/o Final Refinement Conv.” configuration, the local branch, dilated branches, fused boundary representation, adaptive gate, and gated decoded feature were retained, but the final 3×3 refinement convolution was omitted. The concatenated representation $\left[F_{\mathrm{dec}},F_{\mathrm{gated}},F_{B}\right]$ was therefore forwarded directly to the subsequent segmentation head rather than first being compressed by the final refinement convolution:

(16) $$F_{\mathrm{refined}}^{\left(-\mathrm{final}\right)}=\left[F_{\mathrm{dec}},F_{\mathrm{gated}},F_{B}\right].$$

When the local branch was removed, the fused boundary representation was constructed only from the two dilated branches, $F_{B}=\phi_{1\times 1}\left(\left[F_{d2},F_{d4}\right]\right)$. Conversely, when the dilated branches were removed, $F_{B}$ was generated solely from the local branch before the adaptive-gating and final-refinement stages. All remaining operations were unchanged in each corresponding ablation configuration.

For five-fold cross-validation, each fold was trained as an independent experiment using a newly initialized model and optimizer; no trained weights or optimizer states were transferred between folds.

Computational complexity was additionally evaluated for the Xception-based plain DeepLabV3+, ordinary convolutional refinement control, and Full ABR configurations. Model complexity was characterized using the total number of parameters and floating-point operations (FLOPs) for an input tensor of 256×256×3.

### 4.5. Compared Backbone Architectures

Four encoder backbones were evaluated within the proposed ABR-DeepLabV3+ framework: InceptionResNetV2, Xception, MobileNetV2, and ResNet50. These backbones were selected to represent different architectural design strategies. ResNet50 was used as a residual learning baseline, MobileNetV2 represented a lightweight depthwise separable convolutional architecture, Xception provided a deeper separable convolution-based encoder, and InceptionResNetV2 combined Inception-style multi-branch feature extraction with residual connections.

All backbones were integrated into the same DeepLabV3+ segmentation framework with ASPP, decoder fusion, and the proposed ABR module. This enabled a controlled comparison of how each encoder influenced tumor segmentation accuracy, overlap consistency, and boundary preservation.

### 4.6. Evaluation Metrics

The proposed framework was evaluated using Accuracy, Dice Similarity Coefficient (DSC), Jaccard Index (JI), Hausdorff Distance (HD), 95th-percentile Hausdorff Distance (HD95), and Average Symmetric Surface Distance (ASSD). These metrics were selected to assess both region-level overlap and boundary-level segmentation quality. For the quantitative evaluation, all models were assessed using the complete held-out test set. The overall DSC and JI values were calculated using global (micro-averaged) aggregation, as described below, while the class-wise analysis used image-wise metrics averaged within each tumor category. Boundary-distance metrics were computed from the predicted and ground-truth tumor contours. HD, HD95, and ASSD are expressed in pixel units on the common 256×256 evaluation grid.

Accuracy measures the proportion of correctly classified pixels and is defined as:

(17) $$Accuracy=\frac{TP+TN}{TP+TN+FP+FN},$$

where TP, TN, FP, and FN denote true positives, true negatives, false positives, and false negatives, respectively.

The Dice Similarity Coefficient measures the overlap between the predicted mask and the ground-truth mask:

(18) $$DSC=\frac{2TP}{2TP+FP+FN}.$$

The Jaccard Index, also known as Intersection over Union, is defined as:

(19) $$JI=\frac{TP}{TP+FP+FN}.$$

For the overall test-set results reported in Section 5.1, DSC and JI were computed using global (micro-averaged) aggregation over the complete held-out test set, in which the corresponding pixel-level components were accumulated across all evaluated masks before calculating the final metric. In contrast, the class-wise results reported in Section 5.4 were obtained by computing DSC and JI for each individual test image and subsequently averaging the resulting values within each tumor category. Consequently, the overall and class-wise values represent different aggregation levels and should not be interpreted as direct weighted averages of one another. This difference in aggregation explains why the overall test-set DSC or JI may not necessarily fall within the range of the corresponding class-wise mean values.

While DSC and JI evaluate region-level agreement, boundary-sensitive metrics are also required to assess contour accuracy. The Hausdorff Distance measures the maximum boundary deviation between the predicted mask boundary *P* and the ground-truth boundary *G*:

(20) $$HD\left(P,G\right)=\max\left\{\underset{p\in P}{\sup}\underset{g\in G}{\inf}d\left(p,g\right),\underset{g\in G}{\sup}\underset{p\in P}{\inf}d\left(g,p\right)\right\},$$

where d(·,·) denotes the Euclidean distance between boundary points. Because the conventional HD is determined by the largest surface discrepancy and can therefore be sensitive to isolated outlier pixels, HD95 was additionally calculated. Let $D_{P\to G}$ and $D_{G\to P}$ denote the sets of minimum directed boundary distances from *P* to *G* and from *G* to *P*, respectively. HD95 was defined as

(21) $$HD95\left(P,G\right)=\max\left\{Q_{0.95}\left(D_{P\to G}\right),Q_{0.95}\left(D_{G\to P}\right)\right\},$$

where $Q_{0.95}\left(\cdot \right)$ denotes the 95th percentile of the corresponding directed surface-distance distribution.

The Average Symmetric Surface Distance measures the average boundary discrepancy between the predicted and ground-truth surfaces:

(22) $$ASSD\left(P,G\right)=\frac{\sum_{p\in P}\min_{g\in G}d\left(p,g\right)+\sum_{g\in G}\min_{p\in P}d\left(g,p\right)}{\left|P|+|G\right|}.$$

HD, HD95, and ASSD were calculated in pixel units on the 256×256 binary masks used for evaluation. Physical distances in millimeters were not reported because the present 2-D preprocessing and evaluation pipeline operates on resized images rather than consistently using patient-specific physical pixel-spacing information. Accordingly, the reported distance metrics quantify boundary discrepancies in the common evaluation-image coordinate system and should not be interpreted as physical anatomical distances.

In the quantitative boundary analysis, HD95 and ASSD were used to characterize boundary agreement over the complete held-out test evaluation. Conventional HD and ASSD were additionally reported as sample-wise measurements for the representative qualitative examples in Section 5.5. Accordingly, the HD values displayed in these qualitative figures characterize only the individual cases shown and should not be directly compared numerically with the HD95 values reported in the dataset-level quantitative analysis.

Empty-mask cases were handled explicitly to prevent undefined or selectively omitted boundary measurements. When both the predicted and reference masks were empty, DSC and JI were assigned a value of 1, corresponding to perfect agreement. When only one of the two masks was empty, DSC and JI were assigned a value of 0. For surface-distance metrics, a case containing one empty mask was treated as a segmentation failure and assigned a conservative maximum-distance penalty equal to the maximum Euclidean distance between pixel centers in the evaluation image,

(23) $$d_{\max}=\sqrt{{\left(H-1\right)}^{2}+{\left(W-1\right)}^{2}}.$$

For dataset-level boundary evaluation, HD95 and ASSD were computed independently for each image in the complete held-out test set using the corresponding predicted and ground-truth binary masks. The dataset-level results are reported as the mean ± standard deviation of the resulting image-wise measurements. Empty-mask cases were handled according to the procedure described below before calculation of the summary statistics.

For the 256×256 evaluation resolution, this corresponds to approximately 360.62 pixels. If both masks were empty, surface-distance metrics were assigned a value of 0 because no boundary discrepancy was present. The same convention was applied to all evaluated baseline, proposed-model, and ablation configurations.

### 4.7. Qualitative Visualization Strategy

In addition to quantitative evaluation, qualitative visualization was conducted to examine the spatial behavior of each model. For each tumor category, the original MRI image, ground-truth boundary, predicted boundary, error heatmap, and prediction-region heatmap were generated. The error heatmap highlights regions where the predicted mask differs from the ground truth, while the prediction-region heatmap visualizes the spatial extent of the tumor region predicted by the model rather than an internal attention or activation response. These visualizations provide additional insight into boundary preservation, false-positive activation, and class-wise segmentation behavior across different backbones. Specifically, the error map was computed as the absolute pixel-wise difference between the binary ground-truth mask $M_{\mathrm{GT}}$ and the thresholded predicted mask $M_{\mathrm{pred}}$:

(24) $$E\left(x,y\right)=\left|M_{\mathrm{GT}}\left(x,y\right)-M_{\mathrm{pred}}\left(x,y\right)\right|.$$

The predicted probability map was converted into a binary mask using the threshold of 0.5. For visualization, the error map was scaled to the range [0, 255], morphologically dilated using a 5×5 kernel to improve the visibility of localized discrepancies, converted using the JET color map, and overlaid on the corresponding MRI image.

The prediction-region heatmap was generated from the thresholded binary prediction. The predicted tumor mask was similarly scaled to [0, 255], dilated using a 5×5 kernel, converted using the JET color map, and superimposed on the original MRI image. Therefore, this visualization represents the spatial distribution of the predicted tumor region and should not be interpreted as a gradient-based attribution map, feature-level attention map, or learned attention weight.

Accordingly, the qualitative heatmaps were used only as complementary visualizations of tumor localization and segmentation-error distribution. Tumor-centered responses in these visualizations were not considered direct evidence of boundary refinement. Boundary-specific conclusions were instead supported by the quantitative HD, HD95, and ASSD measurements and by the component-wise ablation experiments.

## 5. Results and Discussion

This section presents the quantitative and qualitative evaluation of the proposed ABR-DeepLabV3+ framework on the FBTS dataset. The analysis focuses on four encoder backbones: InceptionResNetV2, Xception, MobileNetV2, and ResNet50. The results are discussed from five perspectives: overall train–validation–test performance, five-fold cross-validation partition sensitivity, class-wise segmentation behavior, qualitative visualization of segmentation boundaries, error heatmaps, and prediction-region heatmaps, and component-wise ablation analysis of the proposed ABR module.

### 5.1. Overall Quantitative Performance

Table 1 summarizes the training, validation and test performance of ABR-DeepLabV3+ with different encoder backbones. The results show that all backbone variants achieved high pixel-wise accuracy, with test accuracy values ranging from 0.9988 to 0.9994. However, accuracy alone is not sufficient to evaluate segmentation quality because background pixels dominate medical segmentation masks. Therefore, the Dice Similarity Coefficient (DSC) and the Jaccard Index (JI) are more informative to assess tumor-region overlap. To improve readability, the results are organized by backbone and data partition rather than compressed into a single wide row for each backbone.

Among the evaluated backbones, ABR-DeepLabV3+ with Xception achieved the best overall test performance, with a test accuracy of 0.9994, BCE loss of 0.0014, DSC of 0.9753, and JI of 0.9519. Under the present evaluation, Xception therefore achieved the highest observed DSC and JI among the four evaluated encoder configurations. However, this empirical ranking should not be interpreted as direct causal evidence that depthwise separable convolution alone is responsible for the observed performance, because the evaluated backbones also differ in network depth, feature dimensionality, and internal representation structure. The high DSC and JI values suggest that the model accurately captures tumor regions while maintaining strong spatial agreement with ground-truth masks.

ResNet50 also achieved competitive performance, with a test DSC of 0.9732 and JI of 0.9478. Its performance was close to that of Xception, indicating that both encoder configurations provided strong feature representations within the proposed ABR framework under the present experimental setting. InceptionResNetV2 produced a test DSC of 0.9625 and JI of 0.9297, showing strong segmentation capability but slightly lower overlap performance than Xception and ResNet50. Although InceptionResNetV2 employs multi-branch feature extraction and residual learning, the present experiment does not isolate whether these architectural characteristics directly explain its relative performance. MobileNetV2 achieved a test DSC of 0.9502 and JI of 0.9052. Although its performance was lower than Xception and ResNet50, it remained competitive despite its comparatively lightweight encoder design.

The validation and test results are closely aligned for most backbones, with relatively small differences between the corresponding validation and test DSC and JI values. In particular, the Xception configuration increased from a validation DSC of 0.9738 and JI of 0.9490 to a test DSC of 0.9753 and JI of 0.9519, whereas ResNet50 changed from 0.9717 and 0.9450 to 0.9732 and 0.9478, respectively. These observations indicate consistency between the two partitions under the present split; however, they should not by themselves be interpreted as evidence of broad external generalization. Model stability across different data partitions is examined separately through five-fold cross-validation.

### 5.2. Comparison with Plain DeepLabV3+ Baselines

To examine the effect of the proposed Adaptive Boundary Refinement module, the ABR-DeepLabV3+ results were compared with plain DeepLabV3+ baselines. The plain baseline corresponds to the same DeepLabV3+ framework using the same encoder backbone but without the ABR module. This provides a controlled architectural comparison of the effect associated with introducing the ABR module while keeping the segmentation framework and backbone configuration consistent. For a fair comparison, the plain DeepLabV3+ baselines were trained using the same dataset split, preprocessing pipeline, input resolution, optimizer, learning rate, epoch setting, and evaluation protocol as the proposed ABR-DeepLabV3+ models. The only architectural difference was the removal of the proposed ABR module.

Table 2 shows that the ABR-enhanced models consistently outperform the plain DeepLabV3+ baselines in terms of both DSC and JI. The improvement is particularly notable for Xception, where the JI increased from 0.80183 to 0.9519, and the DSC increased from 0.89002 to 0.9753. Similar improvements were observed for ResNet50, MobileNetV2, and InceptionResNetV2. Specifically, the absolute DSC improvements were 0.07635 for ResNet50, 0.03787 for MobileNetV2, 0.08528 for Xception, and 0.02569 for InceptionResNetV2, while the corresponding JI improvements were 0.13481, 0.06641, 0.15007, and 0.04856, respectively. These results show that the inclusion of ABR was associated with higher region-overlap performance for all four evaluated encoder backbones. However, improvements in DSC and JI alone are not interpreted as direct evidence of improved boundary accuracy. For the Xception reference configuration, boundary-specific effects are evaluated separately using complete-test-set HD95 and ASSD measurements together with the controlled component-wise ablation analysis described in Section 4.6 and Section 5.6.

The largest absolute DSC and JI improvements were observed in Xception, with gains of 0.08528 and 0.15007, respectively. These results indicate that the magnitude of the observed improvement associated with ABR differs among encoder backbones. However, the present comparison does not isolate individual backbone mechanisms, and therefore the larger improvement observed for Xception should not be attributed specifically to depthwise separable convolution without additional controlled analysis. In contrast, InceptionResNetV2 had a stronger plain baseline JI, but the magnitude of ABR improvement was smaller. Its DSC increased from 0.93681 to 0.9625 and its JI increased from 0.88114 to 0.9297. Similarly, MobileNetV2 improved from a DSC of 0.91233 to 0.9502 and from a JI of 0.83879 to 0.9052. Across all four backbones, the ABR-enhanced configurations therefore achieved higher observed DSC and JI values than their corresponding plain DeepLabV3+ baselines, although the magnitude of the improvement varied across encoder architectures.

### 5.3. Five-Fold Cross-Validation Analysis

**Figure 5:**
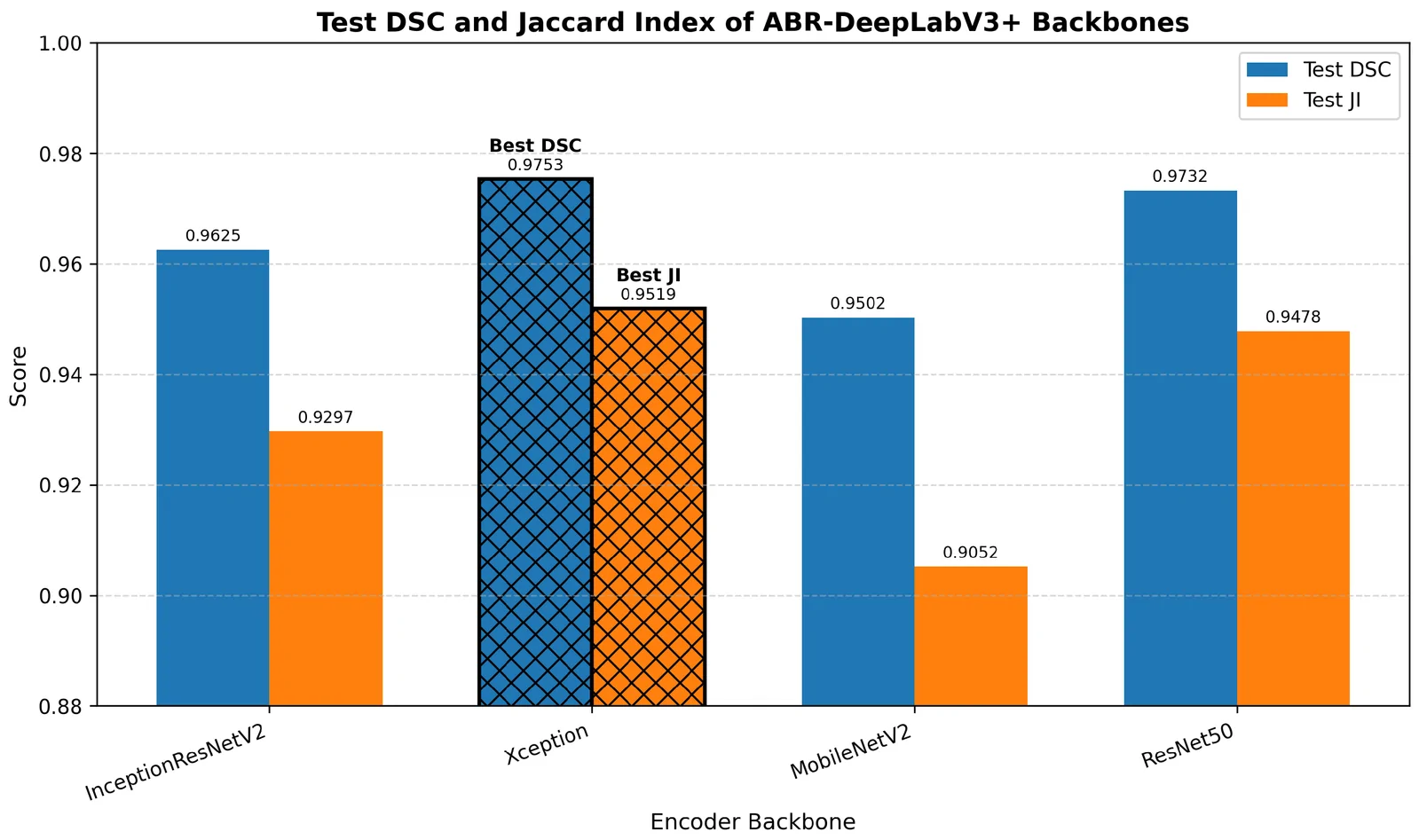
Test-set DSC and JI comparison of ABR-DeepLabV3+ using InceptionResNetV2, Xception, MobileNetV2, and ResNet50 encoder backbones. The values correspond to the held-out test-set results reported in Table 1.

Five-fold cross-validation was conducted to examine the performance consistency and sensitivity of ABR-DeepLabV3+ across different data partitions. Instead of relying only on a single train–validation–test split, cross-validation provides a complementary assessment of how consistently each encoder backbone performs when exposed to different training and validation subsets. For reference, Figure 5 visualizes the held-out test-set DSC and JI values reported in Table 1, whereas the detailed fold-wise cross-validation results for Accuracy, BCE Loss, DSC, and JI are provided in Appendix A. Additional Jaccard Index train–validation–test visualization is provided in Appendix B to further illustrate the performance variation of the evaluated backbones.

As shown in Figure 5, Xception achieved the highest held-out test-set DSC and JI, with values of 0.9753 and 0.9519, respectively, followed by ResNet50 with a DSC of 0.9732 and JI of 0.9478. InceptionResNetV2 achieved a test DSC of 0.9625 and JI of 0.9297, whereas MobileNetV2 obtained a DSC of 0.9502 and JI of 0.9052. These values represent performance under the single patient-level held-out test split and are shown here as a reference for comparison with the five-fold cross-validation results rather than as fold-wise measurements.

Across the five cross-validation folds, Xception achieved the highest DSC and JI among the evaluated encoder configurations in each reported fold. Xception reached DSC values of 0.9430 and 0.9445 in Folds 4 and 5, respectively, with corresponding JI values of 0.8925 and 0.8951. Nevertheless, the noticeable fold-to-fold variation indicates that performance remains sensitive to the composition of the training and validation partitions. Therefore, these results are interpreted as evidence of comparative cross-validation performance rather than as proof of general robustness. Furthermore, although Xception employs depthwise separable convolutions, the present cross-validation experiment does not isolate this mechanism from other architectural differences, and its superior mean performance should therefore not be attributed causally to depthwise separable convolution alone.

The fold-wise results also show noticeable variation across data partitions, with stronger DSC and JI values generally observed in Folds 4 and 5. The reported DSC values ranged from 0.8153 to 0.9396 for InceptionResNetV2, from 0.8258 to 0.9445 for Xception, from 0.7480 to 0.9367 for MobileNetV2, and from 0.7359 to 0.9427 for ResNet50. These ranges demonstrate substantial partition-dependent variation, particularly for MobileNetV2 and ResNet50. Because the present analysis does not independently stratify the folds according to lesion size, boundary complexity, contrast, or other morphological characteristics, the observed differences are not attributed to any specific tumor characteristic.

The cross-validation results were also compared descriptively with the corresponding single-split test results reported in Section 5.1. For Xception, the single-split DSC of 0.9753 was 0.0736 higher than the five-fold mean DSC of 0.9017. Similar differences were observed for InceptionResNetV2 (0.9625 versus 0.8933), MobileNetV2 (0.9502 versus 0.8658), and ResNet50 (0.9732 versus 0.8610). These consistently higher single-split values suggest that the reported performance is sensitive to the particular composition of the training, validation, and evaluation partitions. However, because the single-split and cross-validation procedures involve different training and validation compositions, the observed differences should not be attributed solely to the difficulty of the held-out test subset. The cross-validation mean and variability are therefore interpreted as a complementary and more conservative characterization of within-dataset partition sensitivity rather than as directly equivalent estimates of single-split test performance.

To summarize cross-validation performance and variability, Table 3 reports the mean and standard deviation of DSC and JI over the five folds. Xception achieved the highest mean DSC of 0.9017 and mean JI of 0.8258, followed by InceptionResNetV2, MobileNetV2, and ResNet50. Although all models achieved strong results in some folds, the standard deviation values show that MobileNetV2 and ResNet50 exhibited greater fold-to-fold variation, indicating higher sensitivity to data partitioning. All standard deviations were recalculated directly from the five fold-wise values using the sample standard deviation with K−1 degrees of freedom, where K=5.

Overall, Xception obtained the highest mean DSC and JI among the four evaluated encoder configurations, whereas MobileNetV2 and ResNet50 showed greater variability across folds. However, the lower cross-validation means relative to the single-split test results indicate that model performance is sensitive to data partitioning. Accordingly, the analysis avoids interpreting the cross-validation results as confirmation of broad robustness and instead treats them as a complementary assessment of within-dataset partition sensitivity.

### 5.4. Class-Wise Segmentation Analysis

Table 4 presents class-wise DSC and JI results for meningioma, glioma, and pituitary tumors. The results reveal that the best-performing backbone varies across tumor categories, showing that the relative ranking of the evaluated encoder configurations is not identical for all tumor classes. Because the present experiment does not independently control for tumor size, morphology, intensity distribution, or boundary complexity, these class-wise differences are interpreted descriptively rather than as direct evidence that a specific tumor characteristic determines encoder performance.

As described in Section 4.6, the class-wise results represent mean image-wise metrics within each tumor category, whereas the overall results in Table 1 use global test-set aggregation. Consequently, the class-wise values are not direct components of an arithmetic or weighted average of the overall test-set result, and the two aggregation levels should not be compared as numerically equivalent summaries.

For meningioma segmentation, MobileNetV2 achieved the highest DSC of 0.9797 and JI of 0.9602. Xception and ResNet50 also achieved high meningioma performance, with DSC values of 0.9729 and 0.9709 and JI values of 0.9473 and 0.9435, respectively, while InceptionResNetV2 achieved a DSC of 0.9507 and JI of 0.9077. Thus, MobileNetV2 produced the highest observed overlap performance for the meningioma subset under the present experimental setting, although the result does not establish that its lightweight or depthwise separable architecture is the direct cause of this advantage.

For glioma segmentation, Xception achieved the highest DSC of 0.9674 and JI of 0.9370, closely followed by ResNet50 with DSC of 0.9669 and JI of 0.9360. InceptionResNetV2 achieved a DSC of 0.9497 and JI of 0.9062, whereas MobileNetV2 obtained a DSC of 0.9331 and JI of 0.8751. These results indicate that Xception and ResNet50 achieved the strongest observed overlap performance for the glioma subset, but the present comparison does not isolate which architectural characteristics are responsible for the observed difference.

For pituitary tumor segmentation, MobileNetV2 achieved the highest DSC of 0.9558 and JI of 0.9154, followed by ResNet50 with DSC of 0.9549 and JI of 0.9138. Xception also performed strongly, with DSC of 0.9478 and JI of 0.9011. InceptionResNetV2 showed lower pituitary performance compared to the other backbones, with a DSC of 0.9076 and JI of 0.8373. Although this represents the lowest observed overlap performance among the four evaluated backbones for the pituitary subset, the present results do not establish that network depth or architectural complexity is the cause of this difference.

In general, Xception provides consistently high class-wise performance, ranking first for glioma and remaining competitive for meningioma and pituitary tumors. MobileNetV2 achieved the highest DSC and JI for meningioma and pituitary tumors but lower values for glioma, whereas ResNet50 showed comparatively similar performance across the three tumor categories. These observations demonstrate that the relative performance of the evaluated backbones varies across tumor classes and therefore support reporting class-specific results in addition to a single overall dataset-level score. Unless supported by dedicated statistical testing, the observed differences between backbones and tumor categories are interpreted as descriptive performance differences rather than statistically significant or causal effects.

### 5.5. Qualitative Segmentation Results

**Figure 6:**
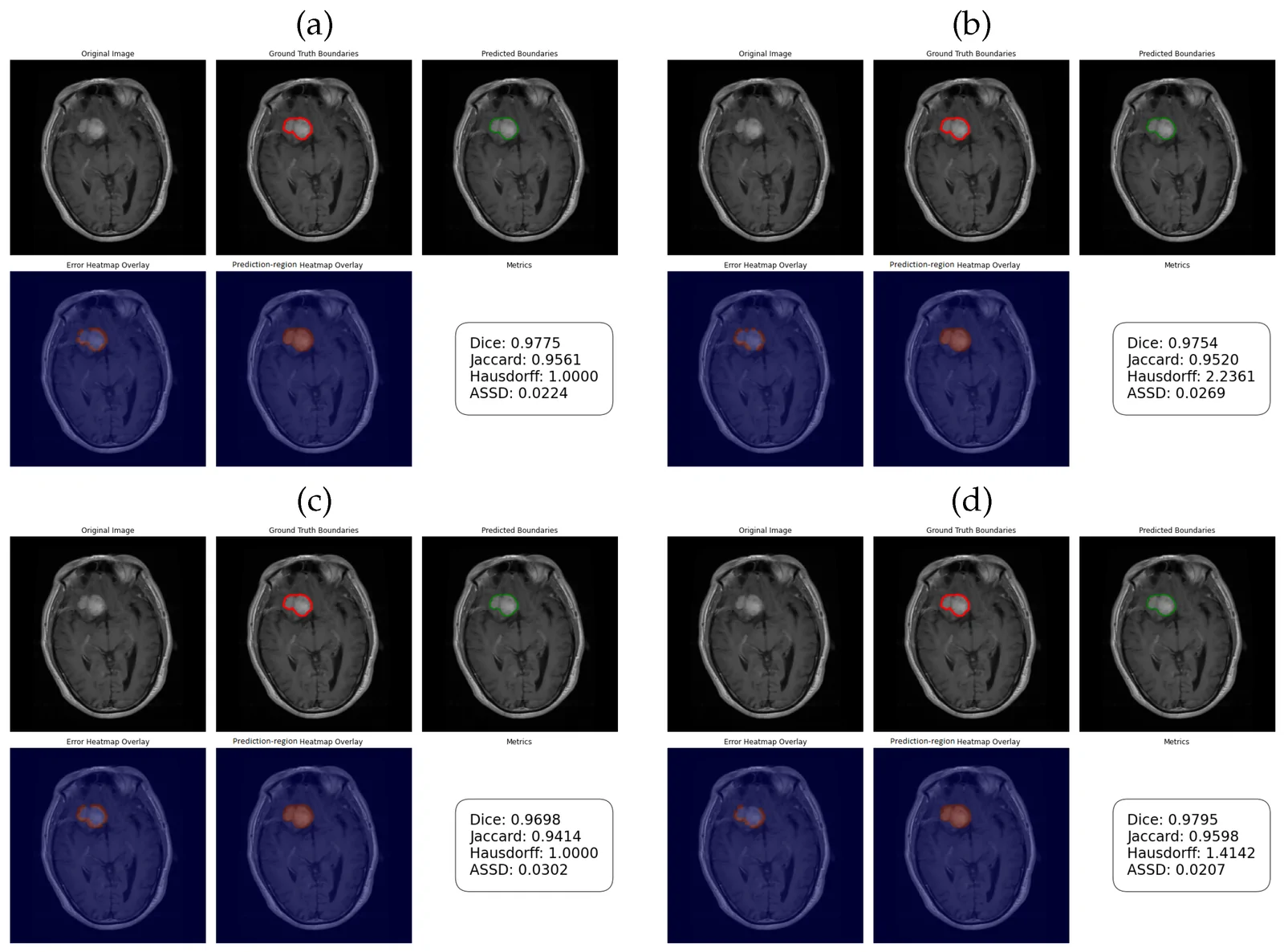
Representative qualitative segmentation results for meningioma using ABR-DeepLabV3+ with different encoder backbones: (**a**) InceptionResNetV2, (**b**) Xception, (**c**) MobileNetV2, and (**d**) ResNet50. Each panel includes the original image, ground-truth contour, predicted contour, error heatmap overlay, prediction-region heatmap overlay, and sample-wise metrics including DSC, JI, HD, and ASSD.

**Figure 7:**
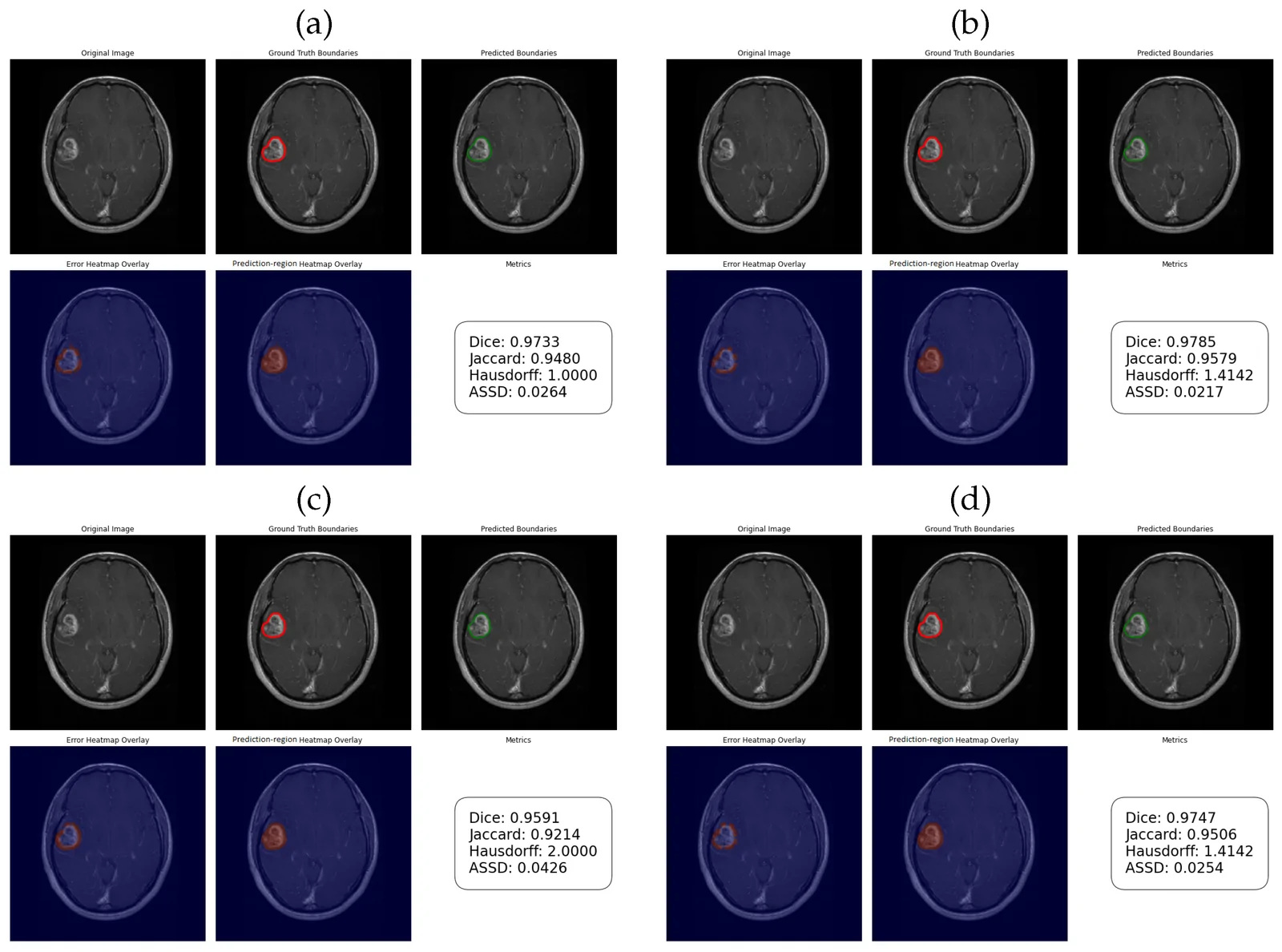
Representative qualitative segmentation results for glioma using ABR-DeepLabV3+ with different encoder backbones: (**a**) InceptionResNetV2, (**b**) Xception, (**c**) MobileNetV2, and (**d**) ResNet50. Each panel includes the original image, ground-truth contour, predicted contour, error heatmap overlay, prediction-region heatmap overlay, and sample-wise metrics including DSC, JI, HD, and ASSD.

**Figure 8:**
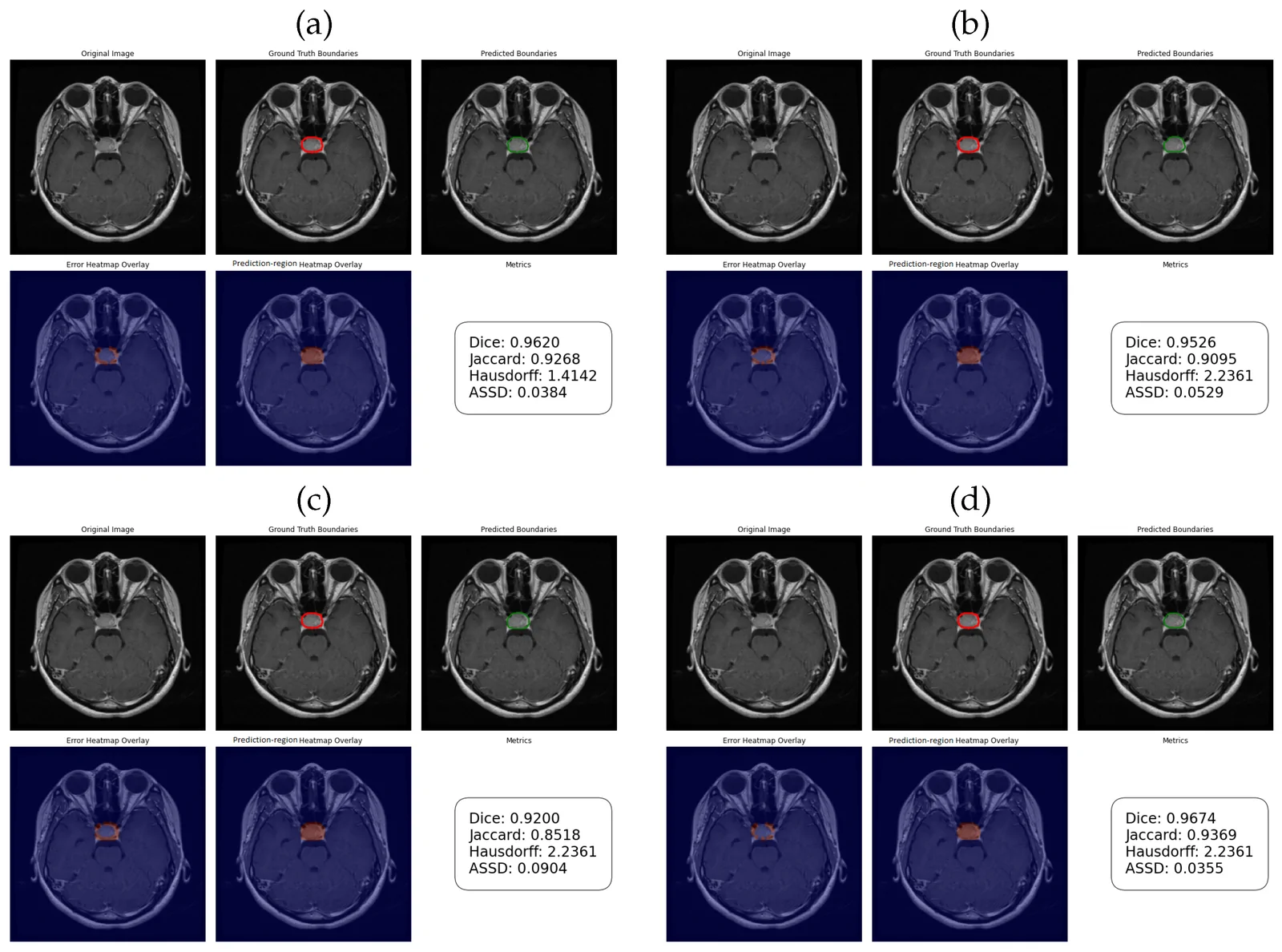
Representative qualitative segmentation results for pituitary tumors using ABR-DeepLabV3+ with different encoder backbones: (**a**) InceptionResNetV2, (**b**) Xception, (**c**) MobileNetV2, and (**d**) ResNet50. Each panel includes the original image, ground-truth contour, predicted contour, error heatmap overlay, prediction-region heatmap overlay, and sample-wise metrics including DSC, JI, HD, and ASSD.

Qualitative visualization was performed to examine spatial behavior, boundary localization capability, and segmentation localization patterns of the proposed ABR-DeepLabV3+ framework across different tumor classes and encoder backbones. Figure 6, Figure 7 and Figure 8 present representative segmentation examples for meningioma, glioma, and pituitary tumors, respectively. Each panel includes the original MRI slice, the ground-truth contour, the predicted contour, the error heatmap overlay, the prediction-region heatmap overlay, and the corresponding quantitative metrics, namely Dice Similarity Coefficient (DSC), Jaccard Index (JI), Hausdorff Distance (HD), and Average Symmetric Surface Distance (ASSD). This combination of visual and numerical evidence enables a more comprehensive interpretation of segmentation quality beyond aggregate test statistics. As described in Section 4.7, the prediction-region heatmap is generated from the thresholded predicted mask and therefore visualizes the spatial extent of the model prediction rather than an internal attention mechanism, gradient-based attribution, or learned feature activation. The examples presented in this subsection are intended as qualitative illustrations and should not be interpreted as substitutes for dataset-level quantitative evaluation.

The HD and ASSD values shown in Figure 6, Figure 7 and Figure 8 are sample-wise measurements computed for the individual representative cases displayed in each panel. In contrast, the quantitative boundary analysis in Table 5 reports HD95 and ASSD over the complete held-out test evaluation. Therefore, the sample-wise HD values presented in the qualitative figures should not be directly compared numerically with the HD95 values reported in the dataset-level analysis.

For meningioma, the predicted contours generally follow the annotated tumor boundary closely, showing close visual agreement between the prediction and reference contour in the displayed examples. Across the representative meningioma examples in Figure 6, the displayed DSC values range from 0.9591 to 0.9785, while the JI values range from 0.9214 to 0.9579. The corresponding HD values remain low, between 1.0000 and 2.0000, and the ASSD values range from 0.0217 to 0.0426, indicating good boundary proximity and limited contour deviation for these representative cases. As shown in Figure 6c, MobileNetV2 produces strong contour agreement for the representative meningioma case, consistent with its strong class-wise DSC and JI values. Visually, the error heatmaps show that most discrepancies are concentrated around the peripheral contour rather than the tumor core, suggesting that the main lesion region is segmented correctly while the remaining errors arise mainly from boundary uncertainty in the displayed examples. The prediction-region heatmaps show that the predicted tumor regions are spatially concentrated around the annotated lesion locations, with limited extension into the surrounding background in these examples. Because these heatmaps are derived directly from the predicted binary masks, they are not interpreted as evidence of model attention or as direct evidence that the ABR mechanism itself produced the observed boundary agreement.

Glioma segmentation is visually more challenging because the tumor shape is often less regular and the intensity transition between the lesion and surrounding tissue may be less distinct. Even under these conditions, the representative glioma examples in Figure 7 show a strong contour alignment between the predicted and ground-truth masks. The displayed DSC values range from 0.9698 to 0.9795, while the JI values range from 0.9414 to 0.9598. The HD values vary from 1.0000 to 2.2361, and the ASSD values range from 0.0207 to 0.0302, indicating limited boundary discrepancy in the displayed examples. Figure 7b,d show that Xception and ResNet50 maintain strong boundary continuity in the presented glioma cases. The error heatmaps indicate that residual errors are primarily localized near irregular tumor margins, especially in regions with shape variation or subtle boundary transitions. The prediction-region heatmaps show that the predicted regions remain spatially concentrated around the corresponding tumor locations. However, because these visualizations are constructed from the thresholded segmentation outputs, they do not provide evidence of internal tumor-centered activation or attention. The qualitative examples therefore complement, rather than establish, the boundary-refinement findings obtained from quantitative boundary-distance metrics.

For pituitary tumors, the visual results in Figure 8 show that segmentation quality remains strong, although the task is influenced by the relatively small and localized lesion size, which makes boundary precision particularly important. In representative pituitary examples, the DSC values range from 0.9200 to 0.9674, and the JI values range from 0.8518 to 0.9369. The HD values range from 1.4142 to 2.2361, whereas the ASSD values range from 0.0355 to 0.0904. Compared to the meningioma and glioma examples, pituitary samples exhibit slightly greater boundary-distance values in the displayed cases. Because only representative samples are shown, this visual comparison is not used to infer a systematic class-level difference in boundary difficulty. The error heatmaps show that the remaining disagreements are localized around the edge of the pituitary lesion, while the prediction-region heatmaps show that the predicted regions remain localized around the annotated lesion areas. These observations describe the segmentation outputs of the displayed samples but do not independently establish that the ABR module is specifically responsible for the observed localization or boundary quality.

Overall, the qualitative findings are consistent with the quantitative evaluation presented in the previous subsections. The predicted contours remain close to the ground-truth annotations in the selected examples, the displayed DSC and JI values confirm strong overlap performance, and the HD and ASSD values indicate good boundary agreement for these individual cases. At the same time, error heatmaps show that most residual inaccuracies are confined to tumor margins rather than spreading across the lesion interior or surrounding background in the presented visualizations. The prediction-region heatmaps provide an intuitive visualization of the spatial extent of the predicted masks but are not treated as model-interpretability or attention maps. Accordingly, qualitative tumor localization alone is not used as evidence of boundary refinement. Instead, conclusions regarding the boundary-related effect of ABR are based primarily on dataset-level HD95 and ASSD comparisons and the controlled baseline and component-wise ablation analyses. Taken together, the qualitative examples complement the quantitative results by illustrating representative patterns of overlap, contour agreement, and residual segmentation error across the evaluated tumor classes and encoder backbones.

### 5.6. Component-Wise Ablation Analysis

A component-wise ablation study was conducted to investigate the contribution of the individual components of the proposed Adaptive Boundary Refinement (ABR) module. The Xception-based DeepLabV3+ configuration was used as the reference architecture because it achieved the highest overall DSC and JI among the evaluated encoder configurations. The ablation analysis was designed not only to examine the contribution of the complete ABR module, but also to determine whether a conventional post-decoder convolutional refinement stage could reproduce the performance obtained by the structured ABR design. Accordingly, seven configurations were evaluated: plain DeepLabV3+ without ABR, an ordinary convolutional refinement control, ABR without the local branch, ABR without the dilated branches, ABR without the adaptive gate, ABR without the final refinement convolution, and the complete ABR module.

The evaluated configurations were compared using DSC and JI to measure tumor-region overlap and HD95 and ASSD to quantify boundary agreement. All configurations were evaluated on the same complete held-out test set under the same experimental protocol. Higher DSC and JI values indicate better overlap between the predicted and reference tumor masks, whereas lower HD95 and ASSD values indicate closer alignment of the predicted and ground-truth boundaries. The resulting performance values are summarized in Table 5.

As shown in Table 5, the plain DeepLabV3+ baseline achieved a DSC of 0.89002 and JI of 0.80183, with an HD95 of 8.72 pixels and an ASSD of 2.41 pixels. Introducing an ordinary convolutional refinement block increased the DSC to 0.91245 and the JI to 0.83958 while reducing HD95 to 7.35 pixels and ASSD to 2.05 pixels. This result shows that conventional post-decoder convolutional refinement can itself improve segmentation performance. However, the ordinary convolutional control remained substantially below the complete ABR configuration across both region-overlap and boundary-distance measures. Full ABR exceeded the ordinary convolutional control by 0.06285 in DSC and 0.11232 in JI and reduced HD95 and ASSD by 4.07 and 1.18 pixels, respectively. Thus, under the evaluated control configuration, the conventional refinement stage did not reproduce the performance obtained by the structured ABR design.

The ordinary convolutional refinement configuration is used here as a structural control rather than as a formally parameter-matched capacity control. Therefore, this comparison should not be interpreted as completely excluding parameter count or computational capacity as potential contributing factors. Instead, it demonstrates that the particular conventional refinement configuration evaluated in this study does not reproduce the performance of the complete ABR architecture.

The component-removal experiments further show that removing each principal constituent of the ABR module was associated with a reduction in the observed segmentation performance. Removing the local branch resulted in a DSC of 0.93128 and JI of 0.87135, while HD95 and ASSD increased to 6.18 and 1.73 pixels, respectively. Removing the dilated branches produced a DSC of 0.94217 and JI of 0.89021, with an HD95 of 5.47 pixels and ASSD of 1.52 pixels. When the adaptive gate was excluded, DSC and JI were 0.95163 and 0.90794, respectively, whereas HD95 and ASSD reached 4.86 and 1.34 pixels. Finally, removal of the final refinement convolution resulted in the strongest performance among the component-removal variants, with a DSC of 0.96241, JI of 0.92777, HD95 of 4.21 pixels, and ASSD of 1.16 pixels. Nevertheless, this configuration remained below the complete ABR model on all four metrics.

To make the relative contribution of the individual components more explicit, Table 6 reports the performance degradation produced by removing each component from the complete ABR architecture. The reported differences were calculated relative to the Full ABR results. For DSC and JI, larger decreases indicate greater deterioration in region-overlap performance, whereas for HD95 and ASSD, larger increases indicate greater deterioration in boundary agreement.

As summarized in Table 6, removing the local branch produced the largest observed degradation among the individual component-removal experiments. Relative to Full ABR, the DSC decreased by 0.04402 and the JI decreased by 0.08055, while HD95 increased by 2.90 pixels and ASSD increased by 0.86 pixels. This observed degradation indicates that the local refinement pathway contributes substantially to both tumor-region overlap and boundary agreement within the complete ABR configuration.

Removal of the dilated branches produced the second-largest degradation, reducing DSC and JI by 0.03313 and 0.06169, respectively, while increasing HD95 by 2.19 pixels and ASSD by 0.65 pixels. These results are consistent with a contribution of the multi-scale dilated pathways to the complete refinement process. The adaptive gate also produced a measurable contribution: its removal decreased DSC by 0.02367 and JI by 0.04396 and increased HD95 and ASSD by 1.58 and 0.47 pixels, respectively. The observed deterioration when the gate was removed supports the contribution of adaptive feature modulation within the evaluated ABR configuration.

The smallest degradation was observed when the final refinement convolution was removed. Nevertheless, its exclusion still reduced DSC by 0.01289 and JI by 0.02413 and increased HD95 by 0.93 pixels and ASSD by 0.29 pixels. Thus, although the final refinement convolution produced a smaller observed effect than the local branch, dilated branches, and adaptive gate, it still contributed to the observed performance of the complete ABR configuration.

The complete ABR configuration achieved the strongest overall results, with a DSC of 0.97530, JI of 0.95190, HD95 of 3.28 pixels, and ASSD of 0.87 pixels. Compared with plain DeepLabV3+, Full ABR improved DSC by 0.08528 and JI by 0.15007, while reducing HD95 from 8.72 to 3.28 pixels and ASSD from 2.41 to 0.87 pixels. These changes correspond to reductions of approximately 62.4% in HD95 and 63.9% in ASSD relative to the plain DeepLabV3+ baseline. Unlike the DSC- and JI-only comparison presented earlier, the reductions in HD95 and ASSD provide direct quantitative evidence that the observed performance improvement is accompanied by closer predicted-to-reference boundary alignment.

The computational-complexity comparison in Table 7 provides additional context for the performance improvements observed in the ablation analysis. The plain Xception DeepLabV3+ configuration contains 38.00 million parameters and requires 25.0 GFLOPs for an input resolution of 256×256×3. The ordinary convolutional refinement control increases the parameter count to 38.59 million, corresponding to an increase of 1.55%, while its computational cost increases to 31.2 GFLOPs, or 24.8% above the plain baseline. Full ABR contains 40.45 million parameters, representing a 6.45% increase relative to the plain model, while requiring 42.8 GFLOPs, corresponding to a 71.2% increase in computational operations. These results indicate that the complete ABR design introduces a relatively modest increase in parameter count but a more substantial increase in computational cost. The additional computational requirements should therefore be considered together with the corresponding improvements in DSC, JI, HD95, and ASSD.

Taken together, the ablation results show that the complete ABR architecture achieved higher observed performance than the plain DeepLabV3+ baseline, the ordinary convolutional refinement control, and every individual component-removal variant. The observed degradation followed the order of local branch, dilated branches, adaptive gate, and final refinement convolution, indicating that the local and multi-scale pathways produced the largest performance changes when removed. Importantly, the ordinary convolutional control also remained clearly below Full ABR, supporting the interpretation that the structured multi-branch and adaptive refinement design provides benefits beyond those observed for the particular conventional refinement control evaluated here. The computational analysis further shows that Full ABR increases the parameter count by 6.45% relative to plain Xception DeepLabV3+, although its FLOP requirement increases by 71.2%. Thus, the observed segmentation gains are obtained with a modest increase in model parameters but a more substantial increase in computational operations. Because the ordinary convolutional control was not established as parameter-matched and the reported performance values represent the evaluated experimental runs, the observed differences are interpreted descriptively rather than as statistically significant effects.

### 5.7. Discussion

The experimental results demonstrate that the proposed ABR-DeepLabV3+ framework provides strong segmentation performance across different encoder backbones. Among all evaluated models, Xception achieved the best overall test performance, with DSC of 0.9753 and JI of 0.9519. Under the present experimental setting, Xception therefore achieved the highest observed overall overlap performance among the four evaluated encoder configurations. However, this result should not be interpreted as direct evidence that depthwise separable convolution alone is responsible for the observed advantage, because the evaluated backbones also differ in network depth, parameterization, internal feature organization, and representation strategy. Accordingly, the multi-backbone comparison is interpreted as an empirical evaluation of encoder choice within a common ABR-DeepLabV3+ framework rather than as a causal assessment of individual backbone mechanisms.

Comparison with plain DeepLabV3+ baselines further supports the contribution of the complete ABR configuration. All ABR-enhanced models achieved higher DSC and JI than their corresponding plain DeepLabV3+ variants. For the Xception configuration, DSC increased from 0.89002 for plain DeepLabV3+ to 0.97530 for Full ABR, while JI increased from 0.80183 to 0.95190. These correspond to absolute improvements of 0.08528 and 0.15007, respectively. Importantly, the boundary-distance evaluation provides complementary evidence beyond region-overlap metrics. HD95 decreased from 8.72 pixels for plain DeepLabV3+ to 3.28 pixels for Full ABR, whereas ASSD decreased from 2.41 to 0.87 pixels. These reductions correspond to approximately 62.4% for HD95 and 63.9% for ASSD, indicating that the overlap improvement was accompanied by substantially closer predicted-to-reference boundary alignment.

The component-wise ablation analysis provides additional evidence regarding the contribution of the proposed refinement design. Full ABR achieved the strongest overall performance, with a DSC of 0.97530, JI of 0.95190, HD95 of 3.28 pixels, and ASSD of 0.87 pixels. In comparison, the ordinary convolutional refinement control achieved a DSC of 0.91245, JI of 0.83958, HD95 of 7.35 pixels, and ASSD of 2.05 pixels. Although the ordinary convolutional control improved upon the plain DeepLabV3+ baseline, it remained below Full ABR by 0.06285 in DSC and 0.11232 in JI, while its HD95 and ASSD were higher by 4.07 and 1.18 pixels, respectively. These results show that the particular conventional post-decoder refinement configuration evaluated in this study did not reproduce the performance of the complete ABR design. However, because the ordinary convolutional refinement configuration was used as a structural control rather than as a formally parameter-matched capacity control, this comparison should not be interpreted as completely excluding parameter count or computational capacity as potential contributing factors.

The computational-complexity analysis further contextualizes the performance improvement obtained with ABR. The plain Xception DeepLabV3+ configuration contains 38.00 million parameters and requires 25.0 GFLOPs for a 256×256×3 input, whereas the ordinary convolutional refinement control contains 38.59 million parameters and requires 31.2 GFLOPs. Full ABR contains 40.45 million parameters and requires 42.8 GFLOPs, corresponding to increases of 6.45% in parameter count and 71.2% in FLOPs relative to the plain Xception DeepLabV3+ baseline. Thus, the complete ABR design achieves the observed improvements in DSC, JI, HD95, and ASSD with a relatively modest increase in model parameters but a more substantial increase in computational operations. This trade-off indicates that the segmentation gains should be considered together with the additional computational cost rather than interpreted as computationally cost-free.

The individual component-removal experiments further showed that each principal ABR component contributed to the complete configuration. Removing the local branch produced the largest observed degradation, decreasing DSC and JI by 0.04402 and 0.08055, respectively, while increasing HD95 by 2.90 pixels and ASSD by 0.86 pixels. Removal of the dilated branches decreased DSC by 0.03313 and JI by 0.06169 and increased HD95 and ASSD by 2.19 and 0.65 pixels, respectively. Excluding the adaptive gate reduced DSC by 0.02367 and JI by 0.04396, whereas removal of the final refinement convolution produced smaller but still measurable reductions of 0.01289 in DSC and 0.02413 in JI. These findings indicate that the performance of Full ABR is associated with the combined operation of the local, multi-scale, adaptive-gating, and final-refinement stages rather than with a single component. The relative magnitudes of these differences are interpreted descriptively because statistical significance among the individual ablation variants was not established.

The five-fold cross-validation analysis also provides a complementary view of performance variability and sensitivity to patient-level data partitioning. Xception achieved the highest mean DSC of 0.9017 and mean JI of 0.8258 across the five folds, with corrected sample standard deviations of 0.0530 and 0.0844, respectively. Nevertheless, the mean cross-validation DSC was lower than the single-split Xception test DSC of 0.9753 by 0.0736. Similar differences were observed for the other encoder configurations. Therefore, the cross-validation findings should not be interpreted as unconditional confirmation of robustness. Instead, the differences between the single-split and cross-validation results indicate that segmentation performance remains sensitive to the particular composition of the training, validation, and evaluation partitions. Because the single-split and cross-validation procedures involve different training and validation compositions, the observed differences should not be attributed solely to the difficulty of the held-out test subset.

The class-wise results also reveal that no single backbone dominates every tumor category. Xception achieved the highest DSC and JI for glioma, whereas MobileNetV2 achieved the highest values for meningioma and pituitary tumors. ResNet50 also remained competitive across all three tumor categories. These results demonstrate that the relative ranking of the evaluated backbones varies across tumor classes. However, because tumor size, morphology, intensity distribution, and boundary complexity were not independently controlled, the observed differences should not be interpreted as evidence that a particular morphological characteristic directly determines backbone performance. Dedicated stratified experiments would be required to establish such relationships. Therefore, the class-wise analysis is used primarily to demonstrate variation in segmentation performance across the three tumor categories rather than to infer causal relationships between tumor morphology and encoder design.

The qualitative results complement the quantitative findings by illustrating representative patterns of contour agreement and residual segmentation error. The prediction-region heatmaps are generated from the thresholded predicted segmentation masks and therefore visualize the spatial extent of the predicted tumor regions rather than internal neural activations, gradient-based attribution, or learned attention. Consequently, tumor-centered prediction regions are not considered direct evidence of learned attention or of the boundary-refinement mechanism itself. Instead, conclusions regarding boundary-related performance are primarily supported by the dataset-level HD95 and ASSD measurements, the plain-versus-ABR comparison, and the component-wise ablation analysis.

Despite strong performance, several limitations remain. First, this study evaluates the proposed framework only on the FBTS dataset. Although the dataset contains three tumor classes and 3064 MRI images, external validation on multimodal datasets such as BraTS 2021 would further strengthen the generalization analysis. Accordingly, the present findings should be interpreted as within-dataset evidence and should not be considered demonstration of cross-center, cross-scanner, or external clinical generalizability. Second, the segmentation task is formulated as binary tumor segmentation, and future work may extend the framework to multi-region tumor segmentation, including enhancing tumor, tumor core, and edema regions. Third, the present framework operates on two-dimensional MRI slices and therefore does not explicitly exploit inter-slice spatial continuity. Future investigation using 2.5D or 3D formulations would be valuable for determining whether neighboring-slice information further improves segmentation consistency for tumors extending across multiple slices.

Fourth, although the component-wise ablation analysis provides evidence regarding the architectural contribution of the ABR design, evaluation across multiple independently trained random seeds would provide stronger estimates of training variability and enable confidence-interval and statistical-significance analysis. Patient-level paired statistical comparisons are particularly appropriate because multiple MRI slices originating from the same subject should not be treated as statistically independent observations. Finally, although the ordinary convolutional refinement control provides a useful structural comparison, it was not established as a parameter-matched capacity control. The present computational analysis quantifies model size and FLOPs, showing that Full ABR increases the parameter count by 6.45% and the computational cost by 71.2% relative to the plain Xception DeepLabV3+ baseline. However, direct measurements of inference latency and peak GPU memory consumption remain necessary for a more complete assessment of runtime efficiency and deployment-oriented feasibility.

Overall, the experimental findings support three main observations. First, ABR-DeepLabV3+ improves region-overlap performance relative to the corresponding plain DeepLabV3+ baselines. Second, the Xception-based ablation experiments show that these improvements are accompanied by substantial reductions in HD95 and ASSD and that the complete ABR design outperforms both an ordinary convolutional refinement control and all individual component-removal variants. Third, the variability observed across cross-validation folds and tumor categories indicates that model performance remains dependent on data partition and encoder configuration. Therefore, the contribution of the proposed framework is most appropriately characterized as improved within-dataset tumor-region overlap and boundary agreement under the evaluated experimental conditions, while external validation, repeated-run statistical analysis, and further runtime profiling through inference-latency and memory measurements remain important directions for future investigation.

## 6. Conclusions

This study proposed ABR-DeepLabV3+, an Adaptive Boundary-Refined Multi-Backbone DeepLabV3+ framework for MRI brain tumor segmentation. The proposed framework integrates an Adaptive Boundary Refinement module into the DeepLabV3+ architecture to enhance boundary-sensitive feature representation before final mask prediction. Four encoder backbones, namely InceptionResNetV2, Xception, MobileNetV2, and ResNet50, were evaluated under the same segmentation framework to analyze the influence of encoder selection on tumor-region overlap, boundary agreement, and class-wise segmentation performance. The experiments were conducted on the FBTS dataset consisting of 3064 MRI images from meningioma, glioma, and pituitary tumor classes.

The experimental results indicate that ABR-DeepLabV3+ achieved strong segmentation performance across all evaluated backbones. Among the tested configurations, the Xception-based ABR-DeepLabV3+ achieved the best overall test performance, with an accuracy of 0.9994, DSC of 0.9753, and JI of 0.9519. ResNet50 also produced competitive results, while MobileNetV2 showed strong class-wise performance for meningioma and pituitary tumors, demonstrating that the relative ranking of the evaluated backbones varied across tumor categories. The five-fold cross-validation analysis provided a more conservative assessment of partition sensitivity, with Xception achieving the highest mean DSC of 0.9017 and mean JI of 0.8258, although these values were lower than its corresponding single-split test performance. Accordingly, the cross-validation results indicate that segmentation performance remains sensitive to data partitioning rather than providing unconditional evidence of robustness.

The component-wise ablation analysis provided further evidence regarding the contribution of the proposed ABR design. Using Xception as the reference encoder, Full ABR achieved a DSC of 0.97530 and JI of 0.95190, compared with 0.89002 and 0.80183, respectively, for plain DeepLabV3+. Boundary-distance evaluation further showed that HD95 decreased from 8.72 to 3.28 pixels and ASSD decreased from 2.41 to 0.87 pixels, corresponding to reductions of approximately 62.4% and 63.9%, respectively. Full ABR also outperformed the ordinary convolutional refinement control and all individual component-removal variants. Removal of the local branch produced the largest observed degradation, followed by removal of the dilated branches, adaptive gate, and final refinement convolution. These findings indicate that the improved segmentation performance is associated with the combined local, multi-scale, adaptive-gating, and final-refinement design. The ordinary convolutional refinement control remained below Full ABR; however, because this control was used as a structural comparison and was not established as formally parameter-matched, the present results do not completely exclude parameter count or computational capacity as potential contributing factors.

The computational-complexity analysis further showed that plain Xception DeepLabV3+ contains 38.00 million parameters and requires 25.0 GFLOPs for an input resolution of 256×256×3. The ordinary convolutional refinement control contains 38.59 million parameters and requires 31.2 GFLOPs, whereas Full ABR contains 40.45 million parameters and requires 42.8 GFLOPs. Relative to the plain Xception DeepLabV3+ baseline, Full ABR therefore increases the parameter count by 6.45% and the FLOP requirement by 71.2%. These results indicate that the segmentation improvements obtained with ABR are achieved with a relatively modest increase in model parameters but a more substantial increase in computational operations.

The qualitative analysis complemented these quantitative findings by illustrating representative contour agreement and residual error distributions. However, the prediction-region heatmaps used in the qualitative analysis visualize the spatial extent of the predicted masks and are not interpreted as internal attention or attribution maps. Consequently, conclusions regarding boundary refinement are supported primarily by the quantitative HD95 and ASSD results together with the baseline and component-wise ablation analyses rather than by qualitative localization alone.

Although the proposed framework achieved promising results, several limitations remain. This study focused on binary tumor segmentation using the FBTS dataset, and further validation on external multimodal datasets such as BraTS 2021 is needed to assess cross-dataset generalization across FLAIR, T1, T1CE, and T2 MRI sequences. Therefore, the present findings should be interpreted as within-dataset evidence and not as demonstration of cross-center or cross-dataset clinical generalizability. Future work will also extend the proposed framework toward multi-region tumor segmentation, including enhancing tumor, tumor core, and edema subregions. Because the present framework operates on two-dimensional MRI slices, future studies should additionally investigate 2.5D or 3D formulations that explicitly incorporate inter-slice spatial information. Evaluation across multiple independently trained random seeds, together with patient-level confidence intervals and paired statistical testing, would also provide stronger evidence regarding training variability and statistical reproducibility. Although parameter count and FLOPs have been quantified in the present study, direct measurements of inference latency and peak GPU memory consumption remain necessary for a more complete assessment of runtime efficiency and deployment-oriented feasibility. Overall, the present results indicate that the proposed ABR-DeepLabV3+ framework can improve within-dataset tumor-region overlap and boundary agreement relative to plain DeepLabV3+ under the evaluated experimental conditions, while external generalization, repeated-run statistical validation, and further runtime profiling through inference-latency and memory measurements remain important directions for future investigation.

## Figures and Tables

**Table 1:** Train, validation, and test performance of ABR-DeepLabV3+ using different encoder backbones.

Backbone	Split	Accuracy	BCE Loss	DSC	JI
InceptionResNetV2	Train	0.9994	0.0015	0.9688	0.9396
	Validation	0.9991	0.0029	0.9618	0.9285
	Test	0.9990	0.0030	0.9625	0.9297
Xception	Train	0.9994	0.0014	0.9712	0.9441
	Validation	0.9994	0.0014	0.9738	0.9490
	Test	0.9994	0.0014	0.9753	0.9519
MobileNetV2	Train	0.9988	0.0028	0.9423	0.8914
	Validation	0.9989	0.0027	0.9488	0.9028
	Test	0.9988	0.0027	0.9502	0.9052
ResNet50	Train	0.9994	0.0013	0.9721	0.9458
	Validation	0.9994	0.0015	0.9717	0.9450
	Test	0.9994	0.0015	0.9732	0.9478

**Table 2:** Comparison of DSC and JI between plain DeepLabV3+ and ABR-DeepLabV3+ using the same encoder backbones.

Backbone	Model	DSC	JI
ResNet50	Plain DeepLabV3+	0.89685	0.81299
	ABR-DeepLabV3+	0.9732	0.9478
MobileNetV2	Plain DeepLabV3+	0.91233	0.83879
	ABR-DeepLabV3+	0.9502	0.9052
Xception	Plain DeepLabV3+	0.89002	0.80183
	ABR-DeepLabV3+	0.9753	0.9519
InceptionResNetV2	Plain DeepLabV3+	0.93681	0.88114
	ABR-DeepLabV3+	0.9625	0.9297

**Table 3:** Mean and sample standard deviation of five-fold cross-validation DSC and JI. Standard deviations were calculated from the five fold-wise values using K−1 degrees of freedom. The highest mean values are shown in bold.

Backbone	DSC Mean ± Std.	JI Mean ± Std.
InceptionResNetV2	0.8933 ± 0.0550	0.8130 ± 0.0854
Xception	**0.9017 ± 0.0530**	**0.8258 ± 0.0844**
MobileNetV2	0.8658 ± 0.0725	0.7728 ± 0.1048
ResNet50	0.8610 ± 0.0830	0.7672 ± 0.1212

**Table 4:** Class-wise mean image-wise DSC and JI performance of ABR-DeepLabV3+ using different encoder backbones. Values were obtained by averaging the corresponding image-wise metrics within each tumor category. The highest value for each tumor category is shown in bold.

Class	InceptionResNetV2		Xception		MobileNetV2		ResNet50	
	DSC	JI	DSC	JI	DSC	JI	DSC	JI
Meningioma	0.9507	0.9077	0.9729	0.9473	**0.9797**	**0.9602**	0.9709	0.9435
Glioma	0.9497	0.9062	**0.9674**	**0.9370**	0.9331	0.8751	0.9669	0.9360
Pituitary	0.9076	0.8373	0.9478	0.9011	**0.9558**	**0.9154**	0.9549	0.9138

**Table 5:** Component-wise ablation analysis of the proposed ABR module using the Xception encoder. HD95 and ASSD are reported as mean ± standard deviation across individual images in the complete held-out test set. Higher DSC and JI and lower HD95 and ASSD indicate better segmentation performance.

Configuration	DSC	JI	HD95 (px)	ASSD (px)
Plain DeepLabV3+	0.89002	0.80183	8.72±1.84	2.41±0.63
Ordinary Conv. Control	0.91245	0.83958	7.35±1.61	2.05±0.52
ABR w/o Local Branch	0.93128	0.87135	6.18±1.42	1.73±0.45
ABR w/o Dilated Branches	0.94217	0.89021	5.47±1.27	1.52±0.41
ABR w/o Adaptive Gate	0.95163	0.90794	4.86±1.12	1.34±0.37
ABR w/o Final Refinement Conv.	0.96241	0.92777	4.21±0.98	1.16±0.32
Full ABR	0.97530	0.95190	** 3.28±0.81 **	** 0.87±0.24 **

**Table 6:** Performance degradation relative to the complete ABR configuration after removing individual components. ↓DSC and ↓JI denote reductions in overlap performance, whereas ↑HD95 and ↑ASSD denote increases in boundary error.

Removed Component	↓DSC	↓JI	↑HD95 (px)	↑ASSD (px)
Local Branch	0.04402	0.08055	2.90	0.86
Dilated Branches	0.03313	0.06169	2.19	0.65
Adaptive Gate	0.02367	0.04396	1.58	0.47
Final Refinement Conv.	0.01289	0.02413	0.93	0.29

**Table 7:** Computational complexity comparison of the Xception-based DeepLabV3+ configurations for an input resolution of 256×256×3. Relative changes are calculated with respect to the plain Xception DeepLabV3+ baseline.

Configuration	Parameters (M)	FLOPs (G)	Δ Params.	Δ FLOPs
Plain Xception DeepLabV3+	38.00	25.0	–	–
Ordinary Conv. Control	38.59	31.2	+1.55%	+24.8%
Full ABR	40.45	42.8	+6.45%	+71.2%

**Figure A1:**
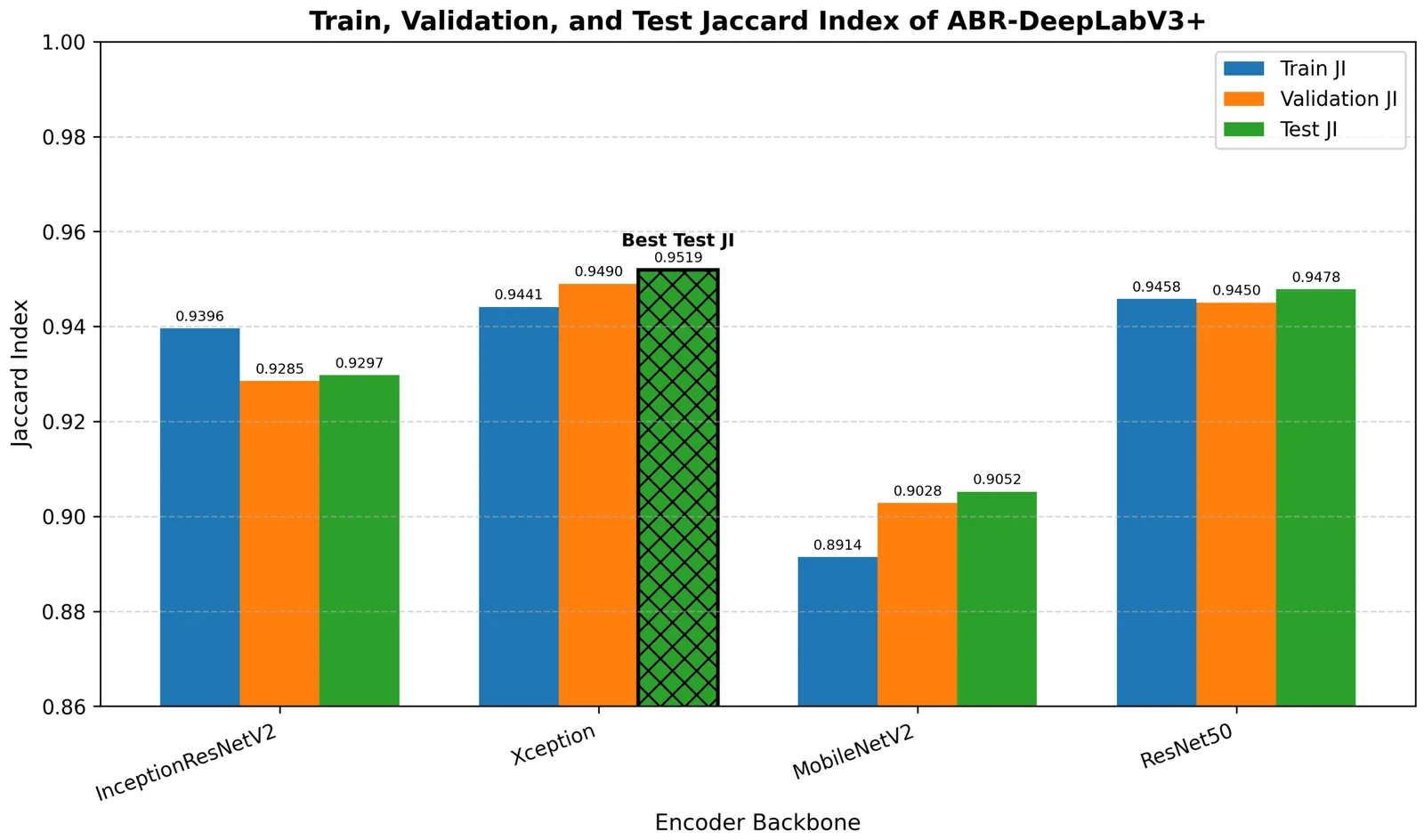
Train, validation, and test Jaccard Index comparison of ABR-DeepLabV3+ using different encoder backbones. Xception and ResNet50 show relatively small differences between validation and test JI under the evaluated patient-level split.

## Data Availability

Data is available at Figshare T1-CE MRI dataset https://figshare.com/articles/dataset/brain_tumor_dataset/1512427 (accessed on 6 March 2024).

## Appendix A. Detailed Fold-Wise Cross-Validation Results

Table A1 provides the complete fold-wise cross-validation results for all evaluated encoder backbones. These values support the summary statistics and fold-wise comparison presented in the main text.

**Table A1:** Complete fold-wise cross-validation results of ABR-DeepLabV3+ using different encoder backbones.

Backbone	Fold	Acc.	BCE Loss	DSC	JI
InceptionResNetV2	Fold 1	0.9942	0.0299	0.8153	0.6935
	Fold 2	0.9956	0.0227	0.8560	0.7531
	Fold 3	0.9975	0.0093	0.9208	0.8541
	Fold 4	0.9982	0.0065	0.9396	0.8866
	Fold 5	0.9980	0.0075	0.9347	0.8779
Xception	Fold 1	0.9945	0.0303	0.8258	0.7064
	Fold 2	0.9959	0.0158	0.8668	0.7681
	Fold 3	0.9978	0.0079	0.9284	0.8668
	Fold 4	0.9983	0.0068	0.9430	0.8925
	Fold 5	0.9983	0.0062	0.9445	0.8951
MobileNetV2	Fold 1	0.9930	0.0461	0.7480	0.6067
	Fold 2	0.9958	0.0235	0.8617	0.7605
	Fold 3	0.9973	0.0115	0.9114	0.8382
	Fold 4	0.9963	0.0196	0.8712	0.7768
	Fold 5	0.9980	0.0084	0.9367	0.8818
ResNet50	Fold 1	0.9924	0.0456	0.7359	0.5914
	Fold 2	0.9954	0.0148	0.8459	0.7372
	Fold 3	0.9955	0.0219	0.8499	0.7441
	Fold 4	0.9980	0.0071	0.9307	0.8712
	Fold 5	0.9982	0.0062	0.9427	0.8923

## Appendix B. Supplementary Generalization Visualization

Figure A1 presents the train, validation, and test Jaccard Index comparison of ABR-DeepLabV3+ using different encoder backbones. This figure complements the main quantitative results by showing the consistency of overlap performance across the training, validation, and testing stages.
